# Ciprofloxacin- and levofloxacin-loaded nanoparticles efficiently suppressed fluoroquinolone resistance and biofilm formation in *Acinetobacter baumannii*

**DOI:** 10.1038/s41598-024-53441-1

**Published:** 2024-02-07

**Authors:** Alaa M. Aboelenin, Mohammed El‑Mowafy, Noha M. Saleh, Mona I. Shaaban, Rasha Barwa

**Affiliations:** 1 Department of Microbiology and Immunology, Faculty of Pharmacy, Mansoura University, PO Box 35516, Mansoura, Egypt; 2 Department of Pharmaceutics, Faculty of Pharmacy, Mansoura University, PO Box 35516, Mansoura, Egypt

**Keywords:** Microbiology, Nanoscience and technology

## Abstract

The spread of fluoroquinolone (FQ) resistance in *Acinetobacter baumannii* represents a critical health threat. This study aims to overcome FQ resistance in *A. baumannii* via the formulation of polymeric nanoFQs. Herein, 80 *A. baumannii* isolates were obtained from diverse clinical sources. All *A. baumannii* isolates showed high resistance to most of the investigated antimicrobials, including ciprofloxacin (CIP) and levofloxacin (LEV) (97.5%). FQ resistance-determining regions of the *gyrA* and *parC* genes were the most predominant resistant mechanism, harbored by 69 (86.3%) and 75 (93.8%) of the isolates, respectively. Additionally, plasmid-mediated quinolone resistance genes *aac*(*6*′)*-Ib* and *qnrS* were detected in 61 (76.3%) and 2 (2.5%) of the 80 isolates, respectively. The CIP- and LEV-loaded poly ε-caprolactone (PCL) nanoparticles, F_CIP_ and F_LEV_, respectively, showed a 1.5–6- and 6–12-fold decrease in the MIC, respectively, against the tested isolates. Interestingly, the time kill assay demonstrated that MICs of F_CIP_ and F_LEV_ completely killed *A. baumannii* isolates after 5–6 h of treatment. Furthermore, F_CIP_ and F_LEV_ were found to be efficient in overcoming the FQ resistance mediated by the efflux pumps in *A. baumannii* isolates as revealed by decreasing the MIC four-fold lower than that of free CIP and LEV, respectively. Moreover, F_CIP_ and F_LEV_ at 1/2 and 1/4 MIC significantly decreased biofilm formation by 47–93% and 69–91%, respectively. These findings suggest that polymeric nanoparticles can restore the effectiveness of FQs and represent a paradigm shift in the fight against *A. baumannii* isolates.

## Introduction

*Acinetobacter baumannii* is an aerobic, non-motile Gram-negative coccobacillus that is considered one of the most hazardous opportunistic pathogens. This organism can get resistance determinants as a result of its genome plasticity, making the infections it causes difficult to treat^1^. The World Health Organization (WHO) has recognized *A. baumannii* as one of the top three priority pathogens requiring urgent development of new antimicrobials. Such dangerous pathogens have the potential to cause several diseases, including post-neurosurgical meningitis, osteomyelitis, lung infections, urinary tract infections, and infections of traumatic or surgical wounds^2^.

*A. baumannii* has developed extraordinary antimicrobial resistance mechanisms, including activated multidrug efflux pumps, increased outer membrane permeability, enzymatic modification of drugs, and target gene mutation. The combined actions of those mechanisms have led to the development of multiple drug-resistant (MDR) and extensively drug-resistant (XDR) strains of *A. baumannii*^3^. XDR is identified as resistance to at least one agent in all but bacterial isolates remain susceptible to one or two categories^4^.

Fluoroquinolones (FQs), such as ciprofloxacin (CIP) and levofloxacin (LEV), have been widely utilized to treat *A. baumannii* infections by inhibiting DNA gyrase and topoisomerase IV. FQ resistance is mostly caused by chromosomal mutations in the fluoroquinolone resistance-determining regions (FQRDRs) of the DNA gyrase genes (*gyrA* and *gyrB*) and/or topoisomerase IV genes (*parC* and *parE*), which reduce DNA gyrase or topoisomerase’s affinity for FQs^5^. Additionally, mutations in the regulatory genes that manage the expression of efflux pumps and outer membrane proteins (OMPs) are considered an important cause of FQ resistance. Plasmid-mediated quinolone resistance (PMQR) plays a crucial role in the acquisition of resistance to FQs. The PMQR genes aid in the selection of mutants with higher levels of resistance rather than conferring FQ resistance^6^. There are different types of PMQR determinants; *qnr* shields FQ targets (bacterial DNA gyrase and topoisomerase IV) from inhibition. *qnr* proteins have been identified, including *qnrS, qnrA, qnrB, qnrD, qnrC, qnrVC*, and the recently discovered *qnrE,* with numerous genetic variants^7^. The second PMQR gene is *aac *(*6*′)*-Ib.* It is an aminoglycoside-modifying enzyme that transfers acetyl groups to some FQs, causing a decrease in binding to the target site and the elimination of antibacterial effects^8^. The enhanced efflux pumps produced by plasmid genes for pumps *qepA* and *oqxAB* are another important mechanism of PMQR^9^.

Biofilms offer an impenetrable barrier to antibacterial agents, providing the necessary conditions for bacterial growth and colonization as well as the emergence of serious and health-threatening microbial infections. Additionally, pathogenic bacteria form biofilms that are encased in the exopolysaccharide matrix, and play a significant role in pathogenesis, and limit the effectiveness of available treatments^10^. As a result, antibacterial therapy requires the search for efficient and biofilm-preventing bactericidal drugs^11^. A smart delivery system has the potential to improve the bactericidal effectiveness of existing antimicrobials and provide an effective solution to combat the spread of resistant bacteria^12^. Developing new generations or derivatives of antimicrobials is a very expensive investment process that takes a long time to distinguish in pharmaceutical production pipelines. Nanosize carriers could provide the necessary chemical protection and the efficient delivery of antimicrobial compounds^12^. This has drawn attention to their potential for preventing and eradicating biofilm development, and microbial resistance. Along with their ability to increase bacterial uptake, antimicrobial-loaded nanoparticles have greater penetration power, which would help to prevent the emergence of MDR and XDR. Additionally, they have greater in vivo stability against biodegradation and require low therapeutic doses and less frequent administration^13^.

Polymeric nanoparticles (NPs) are solid colloidal nano-based systems ranging in size from 10 to 1000 nm. Biodegradable polymers like alginate, chitosan, and polycaprolactone are commonly utilized for the preparation of NPs. Poly ε-caprolactone (PCL) is a semicrystalline aliphatic polyester that degrades at a slower rate than other biodegradable polymers. Such a property can be exploited to deliver antibiotics in a controlled manner over time^14^. Unlike other polyesters, PCL degradation products do not elevate the acidity of the surrounding environment with a minimum impact on homeostasis. PCL was chosen for the preparation of biodegradable NPs in the current study due to its advantageous biocompatibility, biodegradability, and non-toxicity^15^.

The aim of this study is the molecular characterization of different mechanisms of FQ resistance in *A. baumannii* clinical isolates*.* Additionally, CIP- and LEV-loaded polymeric nanoparticles were formulated using PCL and further assessed their effectiveness in overcoming FQ resistance and biofilm formation in *A. baumannii* isolates.

## Results

### Identification of bacterial isolates

#### Microscopical characterization and biochemical reactions

In this study, a total of 550 specimens were collected, and 120 isolates were identified as *Acinetobacter* spp. using standard microbiological techniques, including Gram staining, colony morphology, and biochemical reactions. Under the microscope, all 120 isolates of *Acinetobacter* spp. were seen as Gram-negative coccobacilli. On solid media, colonies were smooth, occasionally mucoid, and non-lactose fermenters appeared as pale or beige colonies on MacConkey agar. Metallic reddish colonies were detected using CHROMagar *Acinetobacter* media after overnight incubation at 37 °C. Furthermore, isolates of *Acinetobacter* spp. were positive for catalase and citrate utilization but were negative for indole, oxidase, methyl red, Voges-Proskauer, and lactose fermentation.

#### Identification of *A. baumannii* by PCR

All the microbiologically identified isolates were confirmed to belong to the *Acinetobacter* genus, as revealed by the detection of a 425 base pairs (bp) amplicon corresponding to the *recA* gene (Supplementary Fig. S1). Furthermore, 80 isolates were identified as *A. baumannii* species, given the codes Ab1-80, via detection of the characteristic 208 bp fragment of the 16S–23S rRNA gene intergenic spacer (*ITS*) region in such species (Supplementary Fig. S1).

The 80 isolates were recovered from blood (n = 28, 35%), sputum (n = 26, 32.5%), wounds (n = 22, 27.5%), and urine (n = 4, 5%), as shown in Supplementary Table S1.

#### Determination of antimicrobial susceptibility

**Figure 1:**
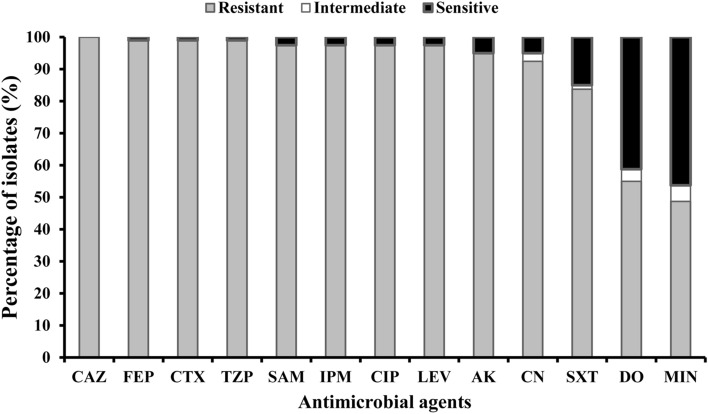
Resistance percentage of the 80 recovered *A. baumannii* isolates to every antimicrobial agent. *CAZ* ceftazidime, *FEP* cefepime, *CTX* cefotaxime, *TZP* tazobactam/piperacillin, *SAM* sulbactam/ampicillin, *IPM* imipenem, *CIP* ciprofloxacin, *LEV* levofloxacin, *AK* amikacin, *CN* gentamicin, *SXT* sulfamethoxazole/trimethoprim, *DO* doxycycline, *MIN* minocycline.

We tested the 80 *A. baumannii* isolates and 2 *A. baumannii* standard strains, ATCC 19606 and ATCC 17987, for susceptibility to β-lactams, FQs, aminoglycosides, a sulfa drug, and tetracyclines using the Kirby–Bauer disc diffusion technique on Mueller–Hinton agar media (Supplementary Table S2). High resistance was detected in all isolates to most of the investigated antimicrobials (Fig. 1). The highest resistance was observed to ceftazidime (100%), cefepime (99%), cefotaxime (99%), tazobactam/piperacillin (99%), sulbactam/ampicillin (97.5%), imipenem (97.5%), ciprofloxacin (97.5%), levofloxacin (97.5%), amikacin (95%), gentamicin (92.5%), and trimethoprim–sulfamethoxazole (83.8%). The lowest level of resistance was observed to doxycycline (55%) and minocycline (48.8%) (Supplementary Table S2). The *A. baumannii* standard strain ATCC 19606 showed sensitivity to all investigated antimicrobials, excluding ceftazidime, cefepime, cefotaxime, tazobactam/piperacillin, sulbactam/ampicillin, and sulfamethoxazole/trimethoprim, while the standard strain ATCC 17987 showed sensitivity to all except sulfamethoxazole/trimethoprim (Supplementary Table S2).

The results demonstrated that 78 isolates (97.5%) were XDR and FQ-resistant isolates (Supplementary Table S2). Eight representative XDR and FQ-resistant isolates (Ab29, Ab30, Ab36, Ab60, Ab65, Ab71, Ab72, and Ab77) were selected to study the effect of the CIP- and LEV-loaded nanopreparations, F_CIP_, and F_LEV_, respectively, on their susceptibility in comparison with free CIP and LEV antimicrobial agents.

### Molecular characterization of FQ resistance mechanisms in *A. baumannii* isolates

#### FQRDRs and target site mutation

**Figure 2:**
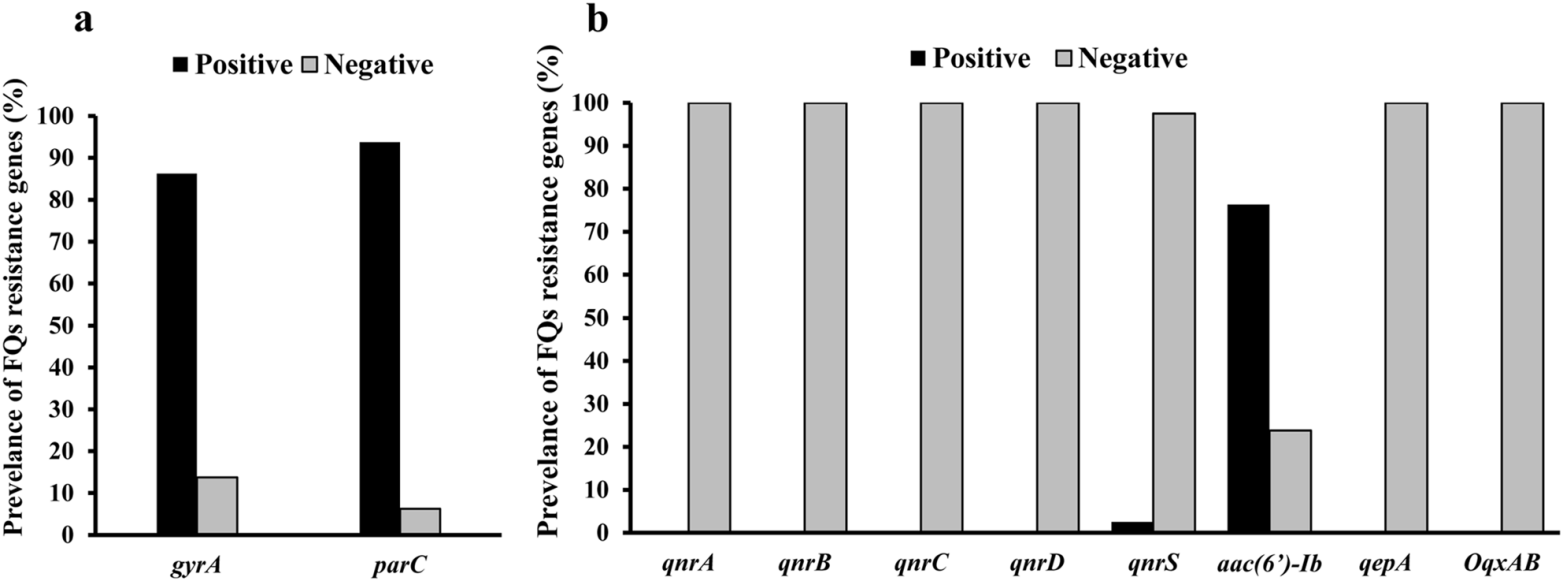
Distribution of fluoroquinolone (FQ) resistance genes among *A. baumannii* isolates; (**a**) fluoroquinolone resistance-determining regions (FQRDRs) and (**b**) plasmid-mediated quinolone resistance genes (PMQRs).

In order to characterize the FQ resistance of the 80 *A. baumannii* isolates and the 2 *A. baumannii* standard strains, the FQRDRs in their genomes were further evaluated. The presence of mutations in *gyrA* and *parC* genes, major FQRDRs, was detected by PCR followed by *HinfI* digestion, which resulted in successful digestion for the PCR products with original sequences but not the ones with mutations (Supplementary Table S3). *gyrA* and *parC* genes were detected in *A. baumannii* with amplicon sizes of 343 bp and 327 bp, respectively. A total of 69 (86.3%) and 75 (93.8%) of the 80 isolates harbored the *gyrA* and *parC* genes, respectively, as shown in Fig. 2a. Non-digested PCR products were obtained after *HinfI* digestion of *gyrA* and *parC* amplicons in all *A. baumannii* isolates, indicating mutations in the FQRDR of both genes (Supplementary Fig. S2). In the two standard *A. baumannii* strains, ATCC 19606 and ATCC17987, obtaining two fragments after *HinfI* digestion, at 291 and 52 bp confirmed the absence of mutation in *gyrA*, while at 206 and 121 bp indicated the absence of mutation in *parC* (Supplementary Table S3).

DNA sequencing of the FQRDRs of *gyrA* and *parC* genes in *A. baumannii* (Ab30, Ab60, and Ab72) isolates showed *gyrA* mutations at Ser83 to Leu and *parC* mutations at Ser80 to Leu. The obtained sequences were deposited in the gene bank with accession numbers OR289925, OR289926, and OR289927.

#### PMQR genes and efflux pump genes

The PMQR genes *qnrA, qnrB, qnrS, qnrC, qnrD, and aac*(*6*′)*-Ib,* as well as efflux pump-encoding genes *oqxAB* and *qepA* were evaluated via PCR (Supplementary Table S3) using the relevant primers shown in Supplementary Table S4. The *aac*(*6*′)*-Ib* and *qnrS* genes were detected in *A. baumannii* with amplicon sizes of 480 bp and 427 bp, respectively (Supplementary Fig. S3). Among 80 *A. baumannii* isolates, the *aac*(*6*′)*-Ib* gene was the most detected PMQR in 61 (76.3%) isolates (Fig. 2b). Two *A*. *baumannii* isolates (2.5%), coded Ab60 and Ab65, harbored the *qnrS* and *aac*(*6*′)*-Ib* genes. Nevertheless, the other PMQR- and efflux pump-encoding genes, *qnrA, qnrB, qnrC, qnrD, qepA,* and *oqxAB,* were absent in all isolates, as shown in Fig. 2b. Additionally, no PMQR genes or efflux pump-encoding genes were detected in the 2 standard *A. baumannii* strains.

#### Preparation and physicochemical evaluation of antimicrobial-loaded PCL nanoparticles

The particle size, polydispersity index (PDI), and zeta potential (ZP) are shown in Table 1. The determination of the above parameters was necessary to assess the capability of the preparation method for producing PCL NPs successfully. The particle size of the prepared NPs was unimodal, ranging from 263.30 ± 2.76 to 271.10 ± 9.14 nm with a very narrow size distribution (PDI ≤ 0.05). The surface of the PCL nanoparticles (NPs) showed a negative charge of approximately 9 mV. The entrapment efficiency (EE%), antibiotic loading (AL%), and yield (Y%) of the NPs are listed in Table 1. CIP was entrapped more efficiently (54.11%) than LEV (28.14%). Consequently, the values of AL% and Y% of F_CIP_ (8.64% and 73.65%, respectively) were higher than their corresponding values for F_LEV_ (5.09% and 64.88%, respectively; Table 1).

**Table 1:** Physicochemical evaluation of CIP- and LEV-loaded nanoparticles.

Parameter	Formula		
	F_CIP_	F_LEV_	Plain NPs
Size (nm)	268.3 ± 4.15	271.1 ± 9.14	263.3 ± 2.76
PDI	0.03 ± 0.01	0.05 ± 0.02	0.03 ± 0.01
ZP (mV)	− 8.88 ± 0.5	− 8.67 ± 0.41	− 8.67 ± 0.9
EE%	54.11 ± 2.66	28.14 ± 4.56	–
AL%	8.64 ± 0.42	5.09 ± 0.83	–
Y%	73.65 ± 12.68	64.88 ± 11.41	66.7 ± 12.94

*CIP* ciprofloxacin, *LEV* levofloxacin, *F*_*CIP*_ CIP-loaded nanoparticles, *F*_*LEV*_ LEV-loaded nanoparticles, *plain NPs* plain PCL nanoparticles, *PDI* polydispersity index, *ZP* zeta potential, *EE%* entrapment efficiency, *AL%* antibiotic loading, *Y%* yield.

**Figure 3:**
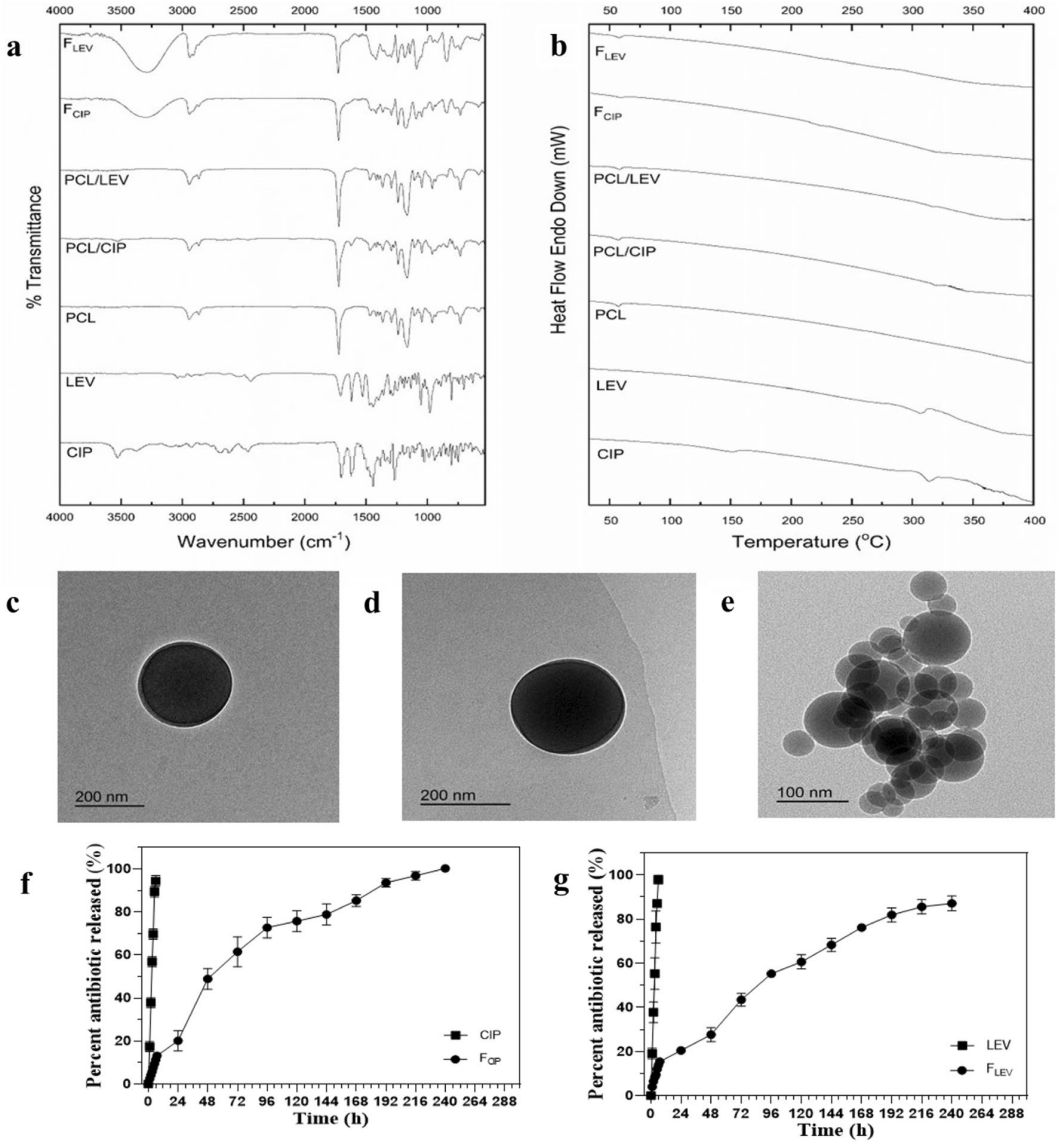
Solid-state characterization of CIP, LEV, PCL, a physical mixture of PCL and CIP, a physical mixture of PCL and LEV, F_CIP_, and F_LEV_; ATR-FTIR (**a**) and DSC (**b**). Morphologies of F_CIP_ (**c**), F_LEV_ and (**d**) Plain NPs (**e**) by TEM and in vitro release of F_CIP_ (**f**) and F_LEV_ (**g**). *CIP* ciprofloxacin, *LEV* levofloxacin, *PCL* poly ε-caprolactone, *F*_*CIP*_ CIP-loaded nanoparticles, *F*_*LEV*_ LEV-loaded nanoparticles, *plain NPs* plain poly ε-caprolactone nanoparticles, *DSC* differential scanning calorimetry, *ATR-FTIR* attenuated total reflectance-Fourier transform infrared spectroscopy, *TEM* transmission electron microscopy.

Solid-state characterization was performed using attenuated total reflectance-Fourier transform infrared spectroscopy (ATR-FTIR) and differential scanning calorimetry (DSC). ATR-FTIR was conducted to evaluate any possible chemical interaction between PCL and CIP or LEV. Moreover, the crystallinity or amorphousness of the developed polymeric matrices of F_CIP_ and F_LEV_ was examined using DSC. The spectra and thermograms of the investigated samples are shown in Fig. 3a,b, respectively. Both CIP and LEV spectra presented characteristic peaks at 1700 (C=O acid), 1620 (C=O carbonyl), 2850–2930 (aromatic H), and 1510–1530 cm^−1^ (piperazinyl group). The PCL spectrum showed characteristic peaks at 1723 (C=O), 1239, 1165 (C–O–C), 2865, and 2944 cm^−1^ (C–H). The spectra of the binary physical mixtures, PCL/CIP and PCL/LEV illustrated the peaks of the polymer, while the peaks of the antimicrobial appeared with diminished intensities or even vanished. The spectra of F_CIP_ and F_LEV_ demonstrated the disappearance of the characteristic peaks of CIP and LEV, respectively.

Thermograms of the investigated samples are shown in Fig. 3b. The pure CIP was shown to be in crystalline state, as evidenced by an endothermic peak at 150 °C. Additionally, other endothermic peaks of the antimicrobials appeared between 300–320 °C. In the thermograms of PCL and binary mixtures (PCL/CIP and PCL/LEV), an endothermic peak of PCL at approximately 57 °C was observed. Thermograms of the binary mixtures also exhibited the melting peaks of the antimicrobial that appeared with reduced intensity. In contrast, the thermograms of F_CIP_ and F_LEV_ demonstrated the amorphousness of their matrices, with no distinctive melting peaks.

The morphology of F_CIP_, F_LEV,_ and plain NPs was studied using transmission electron microscopy (TEM) as shown Fig. 3c–e, respectively. The results confirmed the formation of perfectly spherical, discrete, and uniform NPs with a smooth surface. Individual NPs of F_CIP_ and F_LEV_ had a dense core of entrapped antimicrobials embedded in the polymeric matrix of PCL (core–shell architecture) which was not the case for plain NPs (monophasic polymeric matrix) due to the absence of antimicrobials. Hence, the size of plain NPs was observed to be smaller than that of the antimicrobial-loaded NPs. However, the actual size of the F_CIP_ and F_LEV_ NPs was consistent with that measured by dynamic light scattering (DLS) (approximately 270 nm).

#### In vitro release study and kinetic modeling

The in vitro release experiment was conducted using the dialysis bag diffusion method. This study was performed to compare the release profiles of F_CIP_ and F_LEV_ to the corresponding ones of free CIP and LEV, respectively. The release behaviors of CIP and LEV are expressed as cumulative percentages released from F_CIP_ and F_LEV_, as shown in Fig. 3f,g, respectively. Rapid release rates of 94.4 ± 2.5% and 97.86 ± 2% were exhibited by free CIP and LEV within 6 h, respectively. In contrast, the controlled release patterns of CIP and LEV from F_CIP_ and F_LEV_ extended for 10 days to reach 100 ± 0.3% and 87.09 ± 3.3%, respectively, without burst release (Fig. 3f,g). The release profiles showed that 20.2% and 20.6% of the loaded-CIP and LEV, respectively, released after 24 h incubation and that were even much lower than free ones.

The release data were fitted to kinetic models. It was found that F_CIP_ and F_LEV_ followed non-Fickian anomalous diffusion with diffusion exponent (n) values of 0.72 and 0.52, respectively. On the other hand, the release of the free antimicrobials followed Case II transport with n values of 1.08 and 0.97, respectively.

#### In vitro antimicrobial activity of the prepared nanoantimicrobials F_CIP_ and F_LEV_

The minimum inhibitory concentration (MIC) values were measured using the broth microdilution technique for F_CIP_ and F_LEV_ compared to CIP and LEV, respectively, and displayed in Table 2. Eight representative XDR- and FQ-resistant *A. baumannii* isolates and 2 *A. baumannii* standard strains were selected. The MIC values of CIP ranged from 32 to 128 μg/ml, and the MIC values of LEV ranged from 8 to 16 μg/ml. The MIC values of F_CIP_ and F_LEV_ ranged from 10.7 to 21.5 and 0.72 to 1.3, respectively. However, the MIC values of CIP, LEV, F_CIP_, and F_LEV_ for *A. baumannii* standard strains, ATCC 19606 and ATCC 17987 were 0.5 and 0.25 μg/ml, respectively. F_CIP_ and F_LEV_ were more effective than CIP and LEV, due to the increased efficiency of CIP and LEV to be delivered into the bacterial cells (Table 2). The MIC of F_CIP_ reduced by 1.5- to 6-fold compared to that of free CIP, while the MIC of F_LEV_ decreased by 6- to 12-fold compared to that of free LEV. At the same time, the plain NPs revealed no activity on bacterial growth, indicating that the antimicrobial effects were only obtained from the encapsulated drug itself (Supplementary Table S5).

**Table 2:** The MICs for F_CIP_ and F_LEV_ compared to CIP and LEV against fluoroquinolone (FQ)-resistant *A. baumannii* isolates and standard strains *A. baumannii* ATCC 19606 and 17978.

Isolate code	MIC* (μg/ml)		Fold decrease in MIC	MIC* (μg/ml)		Fold decrease in MIC
	CIP	F_CIP_		LEV	F_LEV_	
Ab29	32	10.7	3	8	0.72	11
Ab30	32	10.7	3	8	0.72	11
Ab36	32	21.5	1.5	8	0.72	11
Ab60	128	21.5	6	16	1.3	12
Ab65	64	21.5	3	16	1.3	12
Ab71	64	21.5	3	8	1.3	6
Ab72	64	21.5	3	8	1.3	6
Ab77	64	21.5	3	8	1.3	6
ATCC 19606	0.5	0.5	1	0.5	0.5	1
ATCC 17987	0.25	0.25	1	0.25	0.25	1

*MIC* minimum inhibitory concentration, *CIP* ciprofloxacin, *LEV* levofloxacin, *F*_*CIP*_ CIP-loaded nanoparticles, *F*_*LEV*_ LEV-loaded nanoparticles.

*The mean MIC value was reported after experiments were carried out in duplicate.

#### Efflux pump suppression by F_CIP_ and F_LEV_

The efflux pump inhibitor carbonyl-cyanide-m-chlorophenylhydrazone (CCCP) was used on the 8 selected representative *A. baumannii* isolates and 2 standard *A. baumannii* strains. Only 3 out of 8 *A. baumannii* isolates, Ab30, Ab30, and Ab72, of which the efflux mechanism was demonstrated to be one of the FQ-resistant mechanisms were used to study the effect of F_CIP_ and F_LEV_ on efflux activity (Supplementary Table S5). That was confirmed by a four-fold or more reduction in MIC (MIC decrease factor (MDF value of more ≥ 4)) in the case of CIP and LEV, while no change in MIC was observed regarding F_CIP_ and F_LEV_ for the same isolates in the presence of CCCP (Table 3). The plain NPs had no antimicrobial (Supplementary Table S5) when used alone or in combination with free CIP or LEV with or without CCCP (Supplementary Table S5). The standard *A. baumannii* strains, had no efflux activity as no change in their MIC of CIP nor LEV with co-treatment with CCCP (Supplementary Table S5). When we used CCCP with strains (Ab29, Ab36, Ab65, Ab71, and Ab77) which do not exhibit an efflux pump related FQ resistance, the addition of the CCCP did not affect the MIC either with free or encapsulated FQ (Supplementary Table S5). Therefore, the increased killing activity of the PCL nanoparticle-encapsulated CIP and LEV is associated with pump inhibition.

**Table 3:** Effect of CCCP on the MICs of F_CIP_, CIP, F_LEV_ and LEV against three representative XDR isolates of *A. baumannii*.

Isolate code	MIC* (μg/ml)		MDF	MIC (μg/ml)		MDF
	CIP	CIP + CCCP		F_CIP_	F_CIP_ + CCCP	
Ab30	32	8	4	10.7	10.7	1
Ab60	128	16	8	21.5	21.5	1
Ab72	64	16	4	21.5	21.5	1

Isolate code	MIC* (μg/ml)		MDF	MIC (μg/ml)		MDF
	LEV	LEV + CCCP		F_LEV_	F_LEV_ + CCCP	
Ab30	8	2	4	0.72	0.72	1
Ab60	16	4	4	0.17	0.17	1
Ab72	8	2	4	1.3	1.3	1

*MIC* minimum inhibitory concentration, *CIP* ciprofloxacin, *LEV* levofloxacin, *F*_*CIP*_ CIP-loaded nanoparticles, *F*_*LEV*_ LEV-loaded nanoparticles, *MDF* MIC decrease factor, *CCCP* carbonyl-cyanide-m-chlorophenylhydrazone.

*The mean MIC value was reported after experiments were carried out in duplicate.

#### Time killing assay

**Figure 4:**
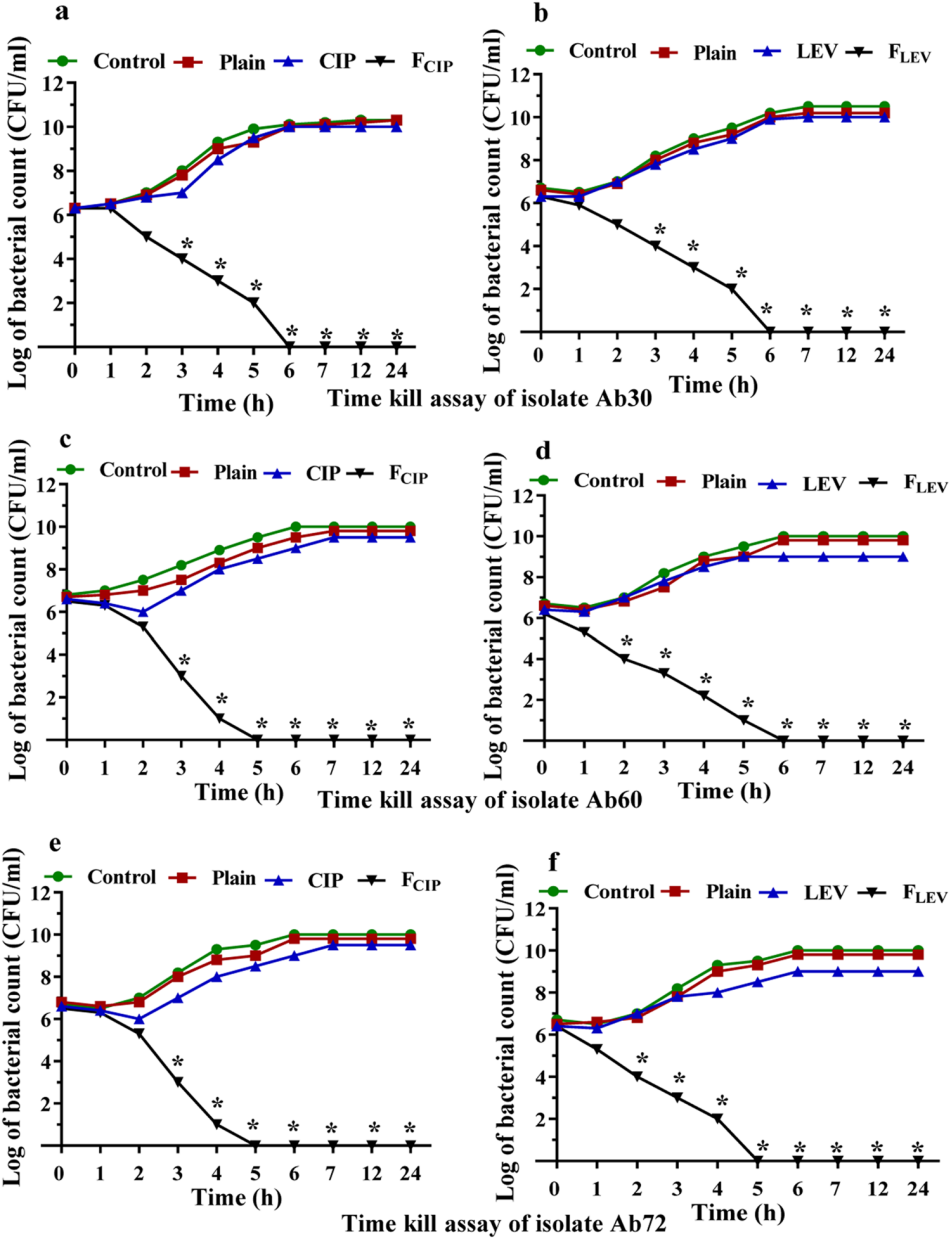
Time kill assay of treated *A. baumannii* isolates with the MIC of F_CIP_ and the MIC of F_LEV_ compared to CIP and LEV over a period of 24 h; *A. baumannii* isolate Ab30 treated with (**a**) F_CIP_ and CIP and (**b**) F_LEV_ and LEV, *A. baumannii* isolate Ab60 treated with (**c**) F_CIP_ and CIP and (**d**) F_LEV_ and LEV and *A. baumannii* isolate Ab72 treated with (**e**) F_CIP_ and CIP and (**f**) F_LEV_ and LEV. Experiments represent three replicates and are expressed as the mean ± SD. Statistical significance was assessed by the one-way ANOVA test: P < 0.05 was considered significant (*P < 0.05). *MIC* minimum inhibitory concentration, *CIP* ciprofloxacin, *LEV* levofloxacin, *F*_*CIP*_ CIP-loaded nanoparticles, *F*_*LEV*_ LEV-loaded nanoparticles, *plain NPs* plain poly ε-caprolactone nanoparticles.

Incubation of *A. baumannii* isolates Ab30, Ab60, and Ab72 with the MICs of F_CIP_ or F_LEV_ caused a significant drop (P < 0.05) in bacterial growth within 2–3 h of incubation and complete bacterial killing after 5–6 h, as shown in Fig. 4. Free LEV reduced the viable bacterial count with 10% inhibition after 24 h incubation at the same concentrations while free CIP showed no effect upon treatment. Treating *A. baumannii* Ab30, Ab60 and Ab72 isolates with the MIC of F_CIP_ caused a significant decrease (P < 0.05) in bacterial growth within 2 h of incubation and complete bacterial killing after 6 h in the Ab30 isolate (Fig. 4a) and after 5 h in the Ab60 and Ab72 isolates, as shown in Fig. 4c,e, respectively. Similarly, the count of such *A. baumannii* isolates significantly reduced after 2 h of incubation with F_LEV_ (P < 0.05), complete bacterial killing after 6 h in the Ab30 and Ab60 isolates (Fig. 4b,d) and after 5 h in the Ab72 isolate, as shown in Fig. 4f. The plain NPs had no killing activity as there was no effect on the viable count of such isolates after 24 h (Fig. 4).

#### Biofilm elimination by F_CIP_ and F_LEV_

**Figure 5:**
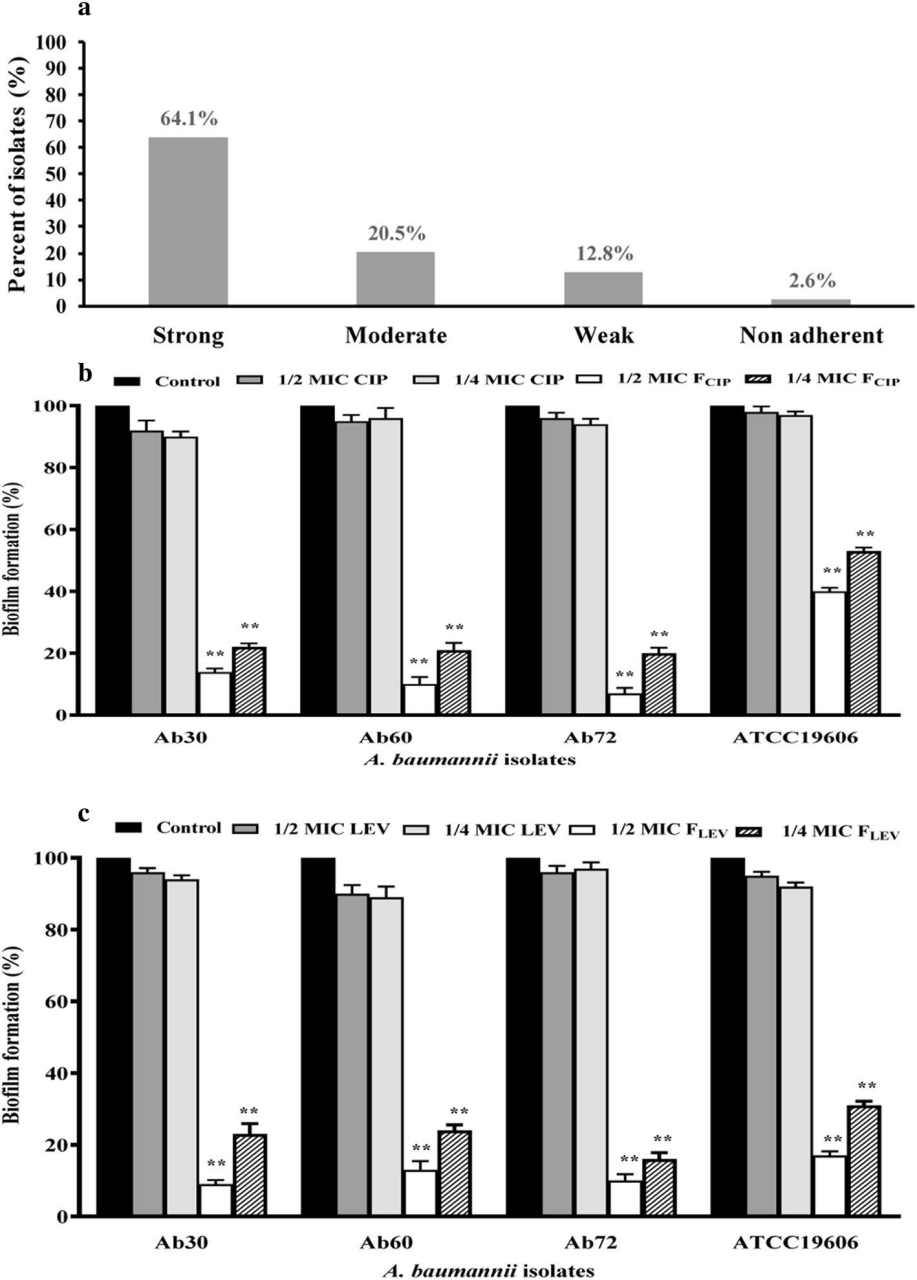
Biofilm formation/inhibition assay; (**a**) Distribution of FQ-resistant *A. baumannii* isolates and standard strains *A. baumannii* ATCC 19606 and 17978 according to biofilm formation. (**b**,**c**) The impact of subinhibitory concentrations (1/2 and 1/4 MIC) of F_CIP_ and F_LEV_ compared to CIP and LEV on biofilm formation of three representative isolates of *A. baumannii* that were both XDR and strong biofilm producers and standard *A. baumannii* strain ATCC19606: (**b**) The effect of F_CIP_ and CIP. (**c**) The effect of F_LEV_ and LEV. Control: Culture treated with plain NPs. Experiments represent four replicates and are expressed as the mean ± SD. Statistical significance was assessed by the one-way ANOVA test: P < 0.01 was considered significant (**P < 0.01). *MIC* minimum inhibitory concentration, *CIP* ciprofloxacin, *LEV* levofloxacin, *F*_*CIP*_ CIP-loaded nanoparticles, *F*_*LEV*_ LEV-loaded nanoparticles, *plain NPs* plain poly ε-caprolactone nanoparticles.

The 80 clinical isolates of *A. baumannii* were classified as strong producers (62.5%, n = 50), moderate producers (21.3%, n = 17) and weak producers (13.8%, n = 11) based on their ability to form biofilms. Only 2.5% (n = 2) of these isolates failed to form biofilms (Supplementary Table S6). The 2 standard *A. baumannii* strains, ATCC 19606 and ATCC17987, were strong biofilm producers (Supplementary Table S6). The results of biofilm adherence of 80 *A. baumannii* isolates are summarized in Supplementary Table S6. Among the 78 FQ-resistant isolates (97.5%), 50 (64.1%) were strong, 16 (20.5%) were moderate, 10 (12.8%) were weak and 2 (2.6%) were non-biofilm forming, as shown in Fig. 5a.

Subinhibitory concentrations of F_CIP_ and F_LEV_ (1/2 and 1/4 MIC) led to a significant dose-dependent decrease in biofilm formation in the selected strong biofilm-forming isolates (Ab30, Ab60, and Ab72) and standard *A. baumannii* strain ATCC19606 compared to cultures treated with CIP and LEV or treated with plain NPs (Fig. 5b,c, respectively). F_CIP_ was associated with 60–93% and 47–80% inhibition in biofilm formation using 1/2 and 1/4 MIC, respectively, in the investigated isolates. Additionally, 1/2 and 1/4 MIC of F_LEV_ caused 83–91% and 69–89% inhibition, respectively, of biofilm formation in such isolates. The greatest reduction (93%) in biofilm formation was achieved using 1/2 MIC F_CIP_ on the Ab72 isolate (Fig. 5b). On the other hand, CIP and LEV were found to be associated with 2–10% and 3–11% inhibition, respectively, when used at their subinhibitory concentrations (1/2 and 1/4 MIC), which indicates that there is no effect of either 1/2 or 1/4 MIC of CIP or LEV on biofilm formation (Fig. 5b,c).

## Discussion

*A. baumannii* is one of the deadliest and most contagious Gram-negative bacteria that has an improved capacity to escape human immune responses and resist several types of antimicrobials, resulting in potentially fatal pneumonia and bacteremia^1^. FQs have been found to be effective against *A. baumannii* isolates over the past 40 years, although resistance to these antimicrobial agents has emerged quickly^2^. There have been several reports of the spread of FQ resistance among *A. baumannii* isolates worldwide^16,17^.

In the present study, 80 isolates of *A. baumannii* were obtained from diverse clinical sources. Among the eighty confirmed *A. baumannii* isolates, resistance to the investigated antimicrobial agents was highly prevalent (Fig. 1). Most of the isolates were XDR (97.5%), while 97.5% of the isolates were resistant to both LEV and CIP. On the other hand, 37 out of 80 isolates were sensitive to minocycline, with the highest frequency (46.3%).

A single point mutation in DNA gyrase is required for *A. baumannii* to be resistant to FQs; nevertheless, simultaneous mutations in the FQRDR areas of the *gyrA* and *parC* genes are anticipated to dramatically increase the level of FQ resistance. Many reports have shown that FQ resistance in *A. baumannii* is related to spontaneous mutations in the FQRDRs of the *gyrA* and *parC* genes^18,19^. The *A. baumannii* isolates were analyzed for their FQRDRs using PCR, followed by *HinfI* digestion. Out of the 80 isolates in this study, 69 (86.3%) and 75 (93.8%) carried the mutations in the *gyrA* (343 bp) and *parC* (327 bp) genes, respectively (Fig. 2a). In *A. baumannii* (Ab30, Ab60, and Ab72) isolates, DNA sequencing of the FQRDRs of the *gyrA* and *parC* genes revealed *gyrA* mutations at Ser83 to Leu and *parC* mutations at Ser80 to Leu. All CIP- and LEV-resistant isolates were found to harbor simultaneous mutations in the FQRDRs of both the *gyrA* and *parC* genes. It is suggested that these two mutations are associated with CIP- and LEV-resistance, as previously mentioned in several reports^20–22^.

PMQR plays a significant role in the development of resistance to FQ and may be a factor in the rise in spontaneous FQRDR mutations^6^. The three most well-known mechanisms of resistance to FQs related to PMQR involve protecting the binding site in DNA-gyrase (*qnr* gene)^7^, altering the drug enzymatically (*aac*(*6*′)*-Ib* gene)^8^, and expelling the agent by efflux pumps (*oqxAB* and *qepA* genes)^9^. Numerous investigations have revealed that FQ-resistant isolates of *A. baumannii* lack *qnrA, qnrB,* and *qnrS* genes^23,24^. Regarding PMQR genes in this study, the *aac*(*6*′)-*Ib* gene (76.3%) was prevalent among the *A. baumannii* isolates, and two isolates (2.5%) harbored the *qnrS* and *aac*(*6*′)*-Ib* genes. However, such isolates lacked other PMQR genes, *qnrA, qnrB, qnrC, qnrD, qepA* and *oqxAB,* as shown in Fig. 2b.

FQ-resistant *A. baumannii* causes serious public and nosocomial infections^16^. Therefore, this study aims to restore the antimicrobial properties of FQs to alter this problem, and polymeric nanoparticles would be the methodology to achieve that. The double emulsification process is the method of choice for encapsulating hydrophilic drugs inside NPs. Although the EE% of the hydrophilic water-soluble drugs is usually low, adjusting the pH values of the internal and external aqueous phases could be applied to enhance the EE%. As FQ contains one carboxylic group and three basic nitrogen sites, its solubility is pH-dependent. At pH 3 of the internal aqueous phase (*W*_*1*_), the soluble cationic species of CIP and LEV predominated. On the other hand, neutral pH stimulates the formation of the zwitterionic least soluble species in the external aqueous phase (*W*_*2*_*,* pH 7.5). Such a preferential solubility pattern could secure efficient loading of the antimicrobials and prevent their diffusion out to the external aqueous phase during the formation of NPs. The higher EE% of F_CIP_ could be attributed to the lower solubility of CIP than LEV at pH 7.5 (*W*_*2*_). Hence, more LEV molecules might prefer their existence in the external aqueous phase rather than entrapping inside the core of the F_LEV_^25^. The negative surface charge of the NPs can be attributed to the ionization of the surface free carboxylic groups of PCL (matrix polymer) in their aqueous dispersion^26^.

The characteristic peaks of CIP, LEV, and PCL in their individual ATR-FTIR spectra in Fig. 3a agree with those reported by other investigators^27,28^. Nevertheless, the diminished intensities of the antimicrobial peaks in the binary physical mixture spectra could be related to the dilution of CIP and LEV with PCL. In the ATR-FTIR spectra of F_CIP_ and F_LEV_, the absence of antimicrobial characteristic peaks indicated their entrapment within the cores of the NPs. However, the bands that appeared in the range of 2800–3500 cm^−1^ were attributed to the characteristic absorption peaks of the hydroxyl groups in polyvinyl alcohol (PVA) (the NP stabilizer).

In the DSC thermogram, the endothermic peaks of the antimicrobials that appeared between 300 and 320 °C in Fig. 3b could be ascribed to the melting of CIP and LEV^29^. The reduction in the intensities of the melting peaks of the antimicrobials exhibited in the thermograms of the binary mixtures could be attributed to the dilution action of PCL. The absence of melting peaks in the thermograms of F_CIP_ and F_LEV_ indicated the amorphousness of the polymeric matrices of the prepared NPs. The findings of the solid-state characterization were consistent with each other. The spherical core–shell morphology of F_CIP_ and F_LEV_ could secure the stealth of antimicrobials. Moreover, the spherical geometry of the NPs could increase bacterial uptake by influencing the contact area with the cell membrane receptors more than rod-shaped particles^30^.

For in vitro release, the sustained performance of F_CIP_ and F_LEV_ in Fig. 3f,g, respectively, might be attributed to the effective entrapment of antimicrobials within their cores. This conclusion agreed with the morphological results, where the compact polymeric coat around the antimicrobial-loaded core likely prolonged antimicrobial release. If the antimicrobial was not efficiently entrapped, an unfavorable initial release (burst release) would have occurred, which was the case with free CIP and LEV. Furthermore, the slow dissolution of the hydrophobic PCL shell of F_CIP_ and F_LEV_ via the hydrolysis of ester bonds followed by pore creation would further prolong the release of the entrapped antimicrobial, which extended for 10 days in our study. This finding could offer the method for the production of antimicrobial with long-lasting activities^31^.

The release data fitted to the kinetic modeling revealed that the release of F_CIP_ and F_LEV_ was ruled mainly by the diffusion mechanism, which ensured a sustained release of the antimicrobials, as observed in their release patterns. Alternatively, the release of free antimicrobials followed Case II transport (zero-order kinetics), which means that the dissolution rather than diffusion was the release limiting step^32^. The latter finding could be attributed to formation of zwitterionic least soluble species of CIP and LEV at pH 7.4 of the release medium^33^. These findings indicate that not only the release pattern but also the release kinetics could be modified by the incorporation of CIP and LEV in PCL-based NPs; F_CIP_ and F_LEV_, respectively.

Numerous studies have indicated improved antibacterial drug activity when entrapped in polymeric nanoparticles^31,34^. These results can be attributed to a variety of variables, such as improved drug delivery to the site of action, increased drug stability when encapsulated into nanoparticles, and easier drug penetration into bacterial cells^35^. In this study, the potency of CIP and LEV loaded into PCL nanoparticles against FQ-resistant *A. baumannii* strains was increased compared to that of free CIP and LEV. Similarly, a CIP polymer-lipid hybrid nanoformulation with greater antibacterial activity against a clinical *E. coli* isolate has been detected^36^.

Indeed, the features of FQs with low molecular weight and zwitterion composition are primarily responsible for their ability to pass through the membrane of Gram-negative bacteria^37^. Additionally, the bacteria have developed resistance to CIP and LEV due to chromosomal mutations that alter the target enzymes. FQ resistance may occur by increasing efflux or decreasing uptake, leading to reduced drug accumulation. Moreover, plasmid-acquired resistance genes can produce proteins protecting bacterial molecules, antimicrobial metabolizing enzymes, or drug efflux pumps^5,6,9^. The MICs of F_CIP_ and F_LEV_ against the investigated *A. baumannii* isolates decreased by 1.5–6- and 6–12-fold, respectively, by encapsulating the drugs into nanoparticles (Table 2). Such a decrease in the MIC levels in our study may be due to enhanced drug penetration by nanoparticles into the bacterial cell, which prevented bacterial development^38^. This behavior can be explained by the fact that NPs can act as carriers for antimicrobials, effectively concealing them, enhancing their penetration of bacterial cell walls, and helping to overcome resistance mechanisms of XDR bacteria^39^. Therefore, the increased membrane permeability obtained by nanosize-encapsulated CIP and LEV may account for the observed improvement in the antibacterial action of F_CIP_ and F_LEV_. Additionally, the prepared particle size affects membrane permeability^40^; therefore, F_CIP_ (268.3 ± 4.15 nm) and F_LEV_ (271.1 ± 9.14 nm) nanoparticles were able to penetrate bacterial cells. The nanosize and charge of the CIP and LEV formulations, as well as the bacterial hydrophobic affinity for the PCL polymer, which aids in rapid permeation across the bacterial outer membrane, may be the cause of the instantaneous microbial killing induced by F_CIP_ and F_LEV_^41^.

FQ resistance in *A. baumannii* isolates may be influenced by efflux-based systems^42^. CCCP increases the sensitivity of several MDR bacteria, including *A. baumannii*, to various antimicrobials by inhibiting efflux pumps^43,44^. A four-fold or greater decline in MIC when CCCP was added to CIP and LEV served as evidence of its significance in increasing CIP and LEV resistance in some isolates^45^. The MICs of F_CIP_ and F_LEV_ nanopreparations were not changed for the same isolates by co-treated CCCP. We thought that the reason for such a result is that the nanoprepared antimicrobials totally inhibited the efflux pump activity, so that the CCCP had no longer effect on the MIC value when added to the culture (Table 3). The efflux-resistant mechanism was defeated by F_CIP_ and F_LEV_, as evidenced by the fact that their MIC was four times lower than that of free CIP and LEV, respectively, on *A. baumannii* isolates with efflux pump activity. NPs can bypass efflux pumps by acting as a Trojan horse, delivering antimicrobials, or by interacting with efflux pumps to create irreversible blockage^31,46^. This notion is strongly supported by the result that MIC for XDR strains without drug efflux pumps was not affected by co-treatment of CCCP with LEV and CIP, either, free or encapsulated into NPs. Similarly, zinc oxide nanoparticles were demonstrated to have a unique efflux pump inhibitory action on *S. aureus* efflux pumps^47^. Additionally, azithromycin poly lactic-co-glycolic acid nanoparticles (AZI-PLGA NPs) found to effectively counter the efflux-resistant mechanism exhibited by AZI-resistant bacteria. This was evidenced by a fourfold decrease in the MIC of NPs compared to free AZI^48^.

The rate of bacterial killing after antimicrobial treatment is critical for preventing the emergence of antimicrobial resistance^34^. The killing activity of CIP and LEV was tremendously enhanced by PCL coating, as revealed by the complete killing of the bacterial cells after 5–6 h of treatment with F_CIP_ and F_LEV_ compared to CIP and LEV, respectively (Fig. 4). On the other hand, after 24 h of incubation at the same concentrations, LEV slightly (10%) decreased the viable bacterial count.

Biofilm is a dispersed microbial growth that is challenging to penetrate and becomes resistant to conventional treatment^10^. A variety of tactics have been studied to improve antibiofilm activity, particularly in relation to biofilms that develop on medical devices that have been implanted. In the present study, 50 of 78 FQ-resistant *A. baumannii* clinical isolates were strong biofilm producers (Fig. 5a). Subinhibitory concentrations of F_CIP_ and F_LEV_ nanopreparations (1/2 and 1/4 MIC) significantly reduced biofilm formation by 47–93% and 69–91% in strong biofilm-forming isolates, whereas CIP and LEV, at their subinhibitory concentrations, affect biofilm formation by 10–17% (Fig. 5b,c). Antimicrobial agents' penetration and effectiveness are improved by the formulation of nanotherapy, which enhances the solubility and minimises the agglomeration of antimicrobials. Nanoformulations of CIP and LEV were found to reduce their particle size and increase their antimicrobial-loaded penetration. Moreover, the hydrophobic properties of PCL chains, which speed up the antimicrobial's penetration and cause the bacterial cell wall to burst, inhibit the growth of biofilms and stop microbial colonization^49^. At the same instance, the effect of metallic nanoparticles on *A. baumannii* biofilms was demonstrated, as they inhibited the biofilm of *A. baumannii* by 88%^50^. Curcumin NPs, aluminium oxide NPs, silver NPs and other nanoparticles were also found to suppress the growth of *A. baumannii* biofilms^51–53^.

In conclusion, this is the first report to study the influence of CIP- and LEV-loaded nanoparticles on FQ resistance and biofilm inhibition of clinical *A. baumannii* isolates in Egypt. The CIP- and LEV-loaded nanoparticles were found to be highly effective in killing *A. baumannii* and inhibiting biofilm formation. Moreover, it was demonstrated that encapsulation of CIP or LEV within NPs was a promising strategy to overcoming efflux-resistant mechanism towards FQs and improve their antibacterial effect. In future work, FQ-loaded PCL nanoparticles will be investigated for efficacy in vivo using adequate animal models.

## Methods

### Materials

Poly ε-caprolactone (PCL, Mn 80,000 kDa), polyvinyl alcohol (PVA, Mw 14 kDa), and methylene chloride (Mw 84.93) were purchased from Aldrich-Sigma Chemical Company, USA. Pharmaceutical grades of ciprofloxacin HCl (CIP) and levofloxacin HCl (LEV) were kindly presented from EPICO and AMOUN pharmaceutical companies in Egypt, respectively. In the nanopreparations procedures, deionized water (Millipore^®^, 18.2 M cm) was used as the source of water.

### Bacterial isolates

*A. baumannii* isolates from clinical specimens were obtained from the Central Microbiology Laboratory of Mansoura University Hospital (MUH), Egypt, between December 2018 and November 2019. These isolates were obtained from diverse clinical specimens from patients in the intensive care unit (ICU), including wounds, sputum, urine, and blood, according to hospital records. This work complies with the ethical guidelines of the Research Ethics Committee in the Faculty of Pharmacy, Mansoura University, Egypt (Permit Number: 2022-193).

*A. baumannii* isolates were purified from the obtained specimens according to standard microbiological culture techniques. All specimens were streaked on the chromogenic culture media *Acinetobacter* (CHROMagar *Acinetobacter* Media, Paris, France) and incubated at 37 °C for 48 h. Isolates were also identified as the *Acinetobacter* genus according to standard microbiological techniques, including colony morphology, Gram stain, and biochemical reactions^54^. The standard strains, *A. baumannii* ATCC 19606 and 17978, were used as positive controls.

### Rapid extraction of genomic DNA, PCR conditions, and purification of PCR products

The genomic DNA of *A. baumannii* isolates was prepared by suspending fresh colonies in 100 µl of distilled water, followed by heating at 95 °C for 10 min. The bacterial suspension was centrifuged at 12,000 rpm for 5 min, and the clear supernatant was transferred to a new tube and stored at − 20 °C.

Unless otherwise specified, Dream Taq polymerase (Fermentas) was used for all routine PCRs. A reaction mixture (25 μl) containing 0.5 μl of each primer (10 μM), 12.5 μl Dream Taq Green PCR Master Mix (2 ×), 1 μl of extracted DNA, and 9.5 μl nuclease-free water was prepared. All PCRs were carried out under the following conditions: primary denaturation at 95 °C for 5 min, followed by 35 cycles of denaturation at 95 °C for 30 s, annealing for 30 s at the temperature specific for each primer pair (Supplementary Table S4), and extension at 72 °C for 1 min, followed by one cycle of final extension at 72 °C for 10 min. The PCR products were analyzed by agarose gel electrophoresis (1.5% w/v agarose gel) and visualized by a UV transilluminator after ethidium bromide staining.

In the case of experiments that required purification of PCR products, digestion of PCR products, and sequencing, a PCR purification kit (Thermo, USA, Catalog number: K0701) was utilized according to the manufacturer’s directions.

### Molecular identification

The isolates were confirmed as *A. baumannii* using a one-tube multiplex PCR method of the *recA* gene (characteristic of the *Acinetobacter* genus) and *ITS* region (specific for *A. baumannii* spp.) by the primers listed in Supplementary Table S4^55^*.* The target amplicons of the *recA* gene and *ITS* region were 425 and 208 bp, respectively. The standard strains, *A. baumannii* ATCC 19606 and 17978, were used as positive controls.

### Detection of antimicrobial susceptibility

The antimicrobial susceptibility profile was determined using the Kirby–Bauer disc diffusion technique on Mueller–Hinton agar media. The following antimicrobial discs (Oxoid, UK) were used to define resistance profiles among *A. baumannii* clinical isolates: ceftazidime (CAZ, 30 µg), cefepime (FEP, 30 µg), cefotaxime (CTX, 30 µg), piperacillin-tazobactam (TZP, 100 μg/10 µg), ampicillin-sulbactam (SAM, 10 μg/10 μg), imipenem (IPM, 10 μg), ciprofloxacin (CIP, 5 μg), levofloxacin (LEV, 5 μg), amikacin (AK, 30 µg), gentamicin (CN, 10 μg), trimethoprim–sulfamethoxazole (SXT, 1.25 μg/23.75 μg), doxycycline (DO, 30 µg) and minocycline (MIN, 30 µg). The inhibition zone diameter was determined and interpreted consistent with the recommendations of the Clinical and Laboratory Standards Institute guidelines^56^.

### Molecular characterization of FQ resistance mechanisms in *A. baumannii* isolates

The FQRDRs of *gyrA* and *parC*, besides the PMQR genes *qnrA, qnrB, qnrS, qnrC, qnrD, and aac*(6′)*-Ib* and the efflux pump-encoding genes *oqxAB* and *qepA,* were amplified via conventional PCR using the relevant primers shown in Supplementary Table S4.

The PCR of the FQRDRs of *gyrA* and *parC* was performed using the specific primers listed in Supplementary Table S4. The purification of amplicons was carried out using a PCR purification kit (Thermo, USA, catalog number: K0701). The purified amplicons were digested with the *HinfI* digestion enzyme (Thermo Scientific, USA) according to the manufacturer’s protocol. The digested PCR products were then separated by agarose gel electrophoresis (1.5% w/v agarose gel). The separated fragments were analyzed for mutations in the FQRDRs^20,21^. Regarding *gyrA,* a single undigested PCR fragment (343 bp) demonstrates the presence of a mutation at Ser83, whereas two fragments, indicating digestion, at 291 and 52 bp confirm the absence of mutation. In the case of *parC,* a single undigested PCR band (327 bp) confirms the presence of a mutation at Ser80, while digestion generating two fragments at 206 and 121 bp indicates the absence of a mutation.

Three representative XDR isolates, Ab30, Ab60, and Ab72, were selected for sequencing of the FQRDRs in both the *gyrA* and *parC* genes. The target sequences in the *gyrA* and *parC* genes were amplified using Phusion High-Fidelity DNA Polymerase (Thermo Scientific, USA) and the specified primers in Supplementary Table S4. PCRs were done according to the manufacturer's instructions. The purified PCR products were sent to Sigma Scientific Service Technical Support Company in Cairo, Egypt for sequencing by an Applied Biosystems 3500 XL Genetic Analyzer and PCR primers specific for each gene. FinchTV program was used to analyze and visualize chromatograms.

### Preparation of NPs

The double emulsion/solvent evaporation method was used to prepare NPs. The ingredients of the prepared NPs are shown in Supplementary Table S7. CIP or LEV was dissolved in sterile deionized water to form *W*_*1*_ at a concentration of 2% w/v. PCL was dissolved in methylene chloride at a concentration of 15 mg/ml to serve as the organic phase (*O*). The pH of *W*_*1*_ was adjusted to 3, and then 1 ml of it was emulsified in 10 ml of *O.* A primary emulsion (*W*_*1*_*/O*) was formed via ultrasonication for 1 min at 100% amplitude in pulse mode (2 s on and 1 s off) (Sonics Vibra Cell, Sonic & Materials, INC, USA) in an ice bath. *W*_*1*_*/O* was added to 200 ml of a pH-adjusted aqueous solution of 0.5% w/v PVA (*W*_*2*_, pH 7.5). This mixture was rapidly sonicated for 3 min under the above conditions to make a double emulsion (*W*_*1*_*/O/W*_*2*_). After solvent evaporation, the NPs were isolated by centrifugation (Benchtop Centrifuge, Sigma Laborzentrifugen, Germany) at 10,000 rpm for 1 h, washed, and centrifuged at the same speed for 30 min to remove a clear supernatant. The NP pellets were dispersed in deionized water then freeze-dried at − 80 °C using a Freeze Dryer (SIM FD8-8T, SIM International, USA). The lyophilized NPs were collected and refrigerated at 4 °C for further evaluation. Plain NPs were prepared by the same procedure as that for drug-free *W*_*1*_ at pH 3.

### Physicochemical evaluation of the prepared NPs

A particle size analyzer (Malvern Instruments Ltd., England) equipped with DLS was used to determine the size and surface charge, or ZP, of the NPs. The EE% of NPs was determined using an indirect method. Briefly, the concentration of unentrapped antimicrobials in the supernatants recovered after centrifugation was measured and subtracted from that of the total antimicrobials. Spectrophotometric measurements of CIP and LEV were conducted using the supernatant of the plain NP as a blank at 275 and 278 nm, respectively (UV–VIS Spectro double beam, Labomed Inc., USA). The weight of lyophilized NPs was determined to calculate the AL% and Y% of NPs. EE%, AL%, and Y% were calculated using the following equations:

$$EE\%=\frac{Total \,Antibiotic - Free\, Antibiotic}{Total\, Antibiotic}\times 100$$

$$AL\%=\frac{Total\, Antibiotic-Free\, Antibiotic}{Wt\, of \, NPs}\times 100$$

$$Y\%=\frac{Wt\, of\, NPs}{Wt\, of\, Antibiotic+PCL}\times 100$$

Spectral analysis of PCL, pure CIP, pure LEV, and binary physical mixtures of each antimicrobial with PCL, F_CIP_, and F_LEV_ was conducted using ATR-FTIR (Thermo Fisher Scientific, Inc., Waltham, MA, USA). DSC was used to assess the crystallinity of the abovementioned samples (DSC, Pyris 6 DSC, Perkin Elmer, USA). The morphology of the NPs was determined by TEM (TEM, JEOL 1010; JEOL Ltd, Tokyo, Japan). Briefly, a 200-mesh copper grid coated with carbon was placed with an aqueous drop of the NP dispersion, and any surplus liquid was absorbed using filter paper. Subsequently, the samples were dried at room temperature so that they could be observed under a 200 kV voltage.

### In vitro release study and kinetic modeling

The in vitro release of CIP and LEV from the NPs was evaluated by the dialysis bag diffusion method. The F_CIP_ or F_LEV_ (equivalent to 2.3 mg) was suspended in 1 ml of deionized water and placed in pre-equilibrated dialysis bags (Dialysis Sacks, Avg. Flat Width 35 mm, MWCO 12 kDa, Sigma-Aldrich). Each dialysis bag was immersed in a beaker containing 100 ml of phosphate buffer (pH 7.4) to represent the release medium and maintained at 37 ± 0.5 °C in a shaking incubator at 100 rpm (GFL Gesellschaft für Labortechnik, Burgwedel, Germany). Every predetermined time interval, 2 ml aliquots were sampled and replaced with an equal fresh volume. The samples were then filtered (0.45 μm), appropriately diluted, and analyzed using spectrophotometry at 275 and 278 nm for CIP and LEV, respectively. Each experiment was done in triplicate, and the cumulative released percentage of the antimicrobial was calculated at every time interval using preconstructed calibration curves. To explain the release mechanisms of CIP and LEV, the release data were kinetically analyzed using the zero-order, first-order, Higuchi diffusion mechanism, and Korsmeyer-Peppas model. The model with the highest correlation coefficient (r^2^) was the one that described the release mechanism.

### Antimicrobial evaluation of the prepared NPs

The activity of F_CIP_ and F_LEV_ against eight representative XDR-resistant isolates was compared to CIP and LEV, respectively. The microtiter plate assay method was used to determine MICs of CIP, LEV and their nanopreparations^56^. Muller-Hinton broth medium (100 μl) was pipetted into sterile microtiter plate wells. CIP, F_CIP_, LEV, and F_LEV_ were prepared as two-fold serial dilutions in Muller-Hinton broth medium ranging from 1024 to 0.5 μg/ml. Inoculation of all dilutions was made with overnight cultures of the isolates at a final inoculum of 5 × 10^5^ CFU/ml. Positive (culture only) and negative (medium only) controls were performed in all experiments. Under the same conditions, the MICs of plain NPs were also determined. The plates were incubated at 37 °C for 24 h. Each experiment was performed in duplicate.

### Effect of F_CIP_ and F_LEV_ on efflux activity

To study the effect of nanoformulated FQs on FQ resistance via an efflux pump mechanism, the MICs of CIP, F_CIP_, LEV and F_LEV_ were determined. The efflux pump inhibitor CCCP was administered at a subinhibitory concentration (20 μg/ml) to each well. The wells were inoculated with diluted culture (5 × 10^5^ CFU/ml) of XDR *A*. *baumannii* isolates (Ab30, Ab60, and Ab72) where the efflux mechanism was demonstrated to be one of the FQ-resistant mechanisms*.* Furthermore, the effect of CCCP on the MICs of plain NPs, CIP, and LEV against representative FQ-resistant and XDR *A. baumannii* isolates and 2 standard strains *A. baumannii* was investigated. A positive control for each isolate was included to investigate the viability of different isolates in the presence of CCCP alone. The MDF was determined for each isolate in duplicate. Inhibition of the efflux pump by CCCP was deemed to have a considerable effect when the MDF value was 4 or above^57^.

### Antimicrobial killing assay

The killing rate of FQ-resistant bacteria by F_CIP_ and F_LEV_ was determined and compared to CIP- and LEV-free antimicrobials, respectively. The investigated XDR *A. baumannii* isolates (Ab30, Ab60, and Ab72) were propagated until the bacterial count reached 5 × 10^6^ CFU/ml. The investigated isolates were treated with F_CIP_ and F_LEV_ at the MIC and incubated at 37 °C. Samples were collected at 0, 1, 2, 3, 4, 6, 10, and 24 h, and each sample was ten-fold serially diluted to determine the viable bacterial count. In the same instance, bacterial counts of cultures treated with CIP and LEV were also performed under the same conditions. As a control, bacterial growth without free antimicrobials (CIP or LEV) or nanoformulated antimicrobials (F_CIP_ or F_LEV_) and plain NPs were also investigated. The surface drop method was used in triplicate to calculate the number of bacteria that recovered over time following treatment^58^. Following antimicrobial treatment, the number of recovered cells was plotted against the CFU/ml over time. Each experiment was carried out in triplicate.

### Effect of F_CIP_ and F_LEV_ on biofilm formation

The capacity of biofilm formation among 80 *A. baumannii* isolates and 2 standard *A. baumannii* strains, ATCC 19606 and ATCC17987, was assessed in vitro using 96-well microtiter plates as previously mentioned^59^. The formed biofilm was stained with 1% w/v crystal violet followed by solubilization using glacial acetic acid (33% v/v). The solubilized biofilm was measured using an ELx808TM Absorbance Microplate Reader (BioTek Instruments Inc., Winooski, VT) at OD_490 nm_. A negative control of the medium was included in each experiment. The mean OD_490 nm_ of each bacterial isolate from four independent experiments was calculated to assess the ability of *A. baumannii* isolates to produce biofilms. Biofilm formation by the *A. baumannii* isolate was repeated in quadruplicate.

To determine the biofilm formation capacity of *A. baumannii* isolates, the cut-off optical density (ODc) was established as three standard deviations above the mean OD of the inoculum-free negative control. Strains were classified as follows: non-biofilm producer (N) if OD ≤ ODc, weak biofilm producer (W) if ODc < OD_W_ ≤ 2 ODc, moderate biofilm producer (M) if 2 ODc < OD_M_ ≤ 4 ODc, and strong biofilm producer (S) if OD_S_ > 4 ODc.

Three representative strong biofilm-forming, FQ-resistant, and XDR isolates (Ab30, Ab60, and Ab72) were selected to assess the effect of F_CIP_ and F_LEV_ on biofilm formation. Subinhibitory concentrations for CIP, F_CIP_, LEV, and F_LEV_ (1/2 and 1/4 MIC) were incorporated during bacterial incubation in 96-well plates^60^. Controls of medium only or cultures without drugs were included in each experiment. The biofilm-forming reference strain of *A. baumannii* ATCC 19606 was used as a positive control.

### Statistical data analysis

Statistical analysis involved calculating the mean and standard deviation of the values. All microbiological assays were performed in duplicate except the antimicrobial killing assay, and the capacity for biofilm formation was repeated in triplicate and quadruplicate, respectively. A one-way ANOVA test was calculated, with the significance value set at P < 0.05 or P < 0.01 using the GraphPad Prism software package (version 8.3.0).

### Ethics approval

The Research Ethics Committee at the Faculty of Pharmacy at Mansoura University in Egypt (Permit Number: 2022-193) has approved this work as ethically compliant.

### Supplementary Information

Supplementary Information.

## Data Availability

All data generated or analyzed during this study are included in this published article [and its supplementary information file].
