# Supplementary material for: Ciprofloxacin- and levofloxacin-loaded nanoparticles efficiently suppressed fluoroquinolone resistance and biofilm formation in Acinetobacter baumannii
Source: Sci Rep. 2024 Feb 7;14:3125. doi: 10.1038/s41598-024-53441-1 (PMC10850473; doi:10.1038/s41598-024-53441-1)
Supplement: Supplementary file 1 — Supplementary Information. [file 41598_2024_53441_MOESM1_ESM.pdf]

Supplementary materials

Ciprofloxacin- and Levofloxacin-Loaded Nanoparticles Efficiently Suppressed Fluoroquinolone Resistance and Biofilm Formation in *Acinetobacter baumannii*

Alaa M. Aboelenin [1], Mohammed El-Mowafy [1], Noha M. Saleh [2], Mona I. Shaaban [1]* and Rasha Barwa [1]**.

[1]Department of Microbiology and Immunology, Faculty of Pharmacy, Mansoura University, PO Box 35516, Mansoura, Egypt
[2]Department of Pharmaceutics, Faculty of Pharmacy, Mansoura University, PO Box 35516, Mansoura, Egypt

*** Correspondence:** mona_ibrahim@mans.edu.eg

****Co- correspondence:** rasha@mans.edu.eg

Supplementary Table S1. *A. baumannii* isolates identified in this study and their clinical sources.

Isolate Code	Clinical source	Isolate Code	Clinical source	Isolate Code	Clinical source
**Ab1**	blood	**Ab28**	blood	**Ab55**	Wound
**Ab2**	blood	**Ab29**	sputum	**Ab56**	Wound
**Ab3**	blood	**Ab30**	sputum	**Ab57**	Wound
**Ab4**	blood	**Ab31**	sputum	**Ab58**	Wound
**Ab5**	blood	**Ab32**	sputum	**Ab59**	Wound
**Ab6**	blood	**Ab33**	sputum	**Ab60**	Wound
**Ab7**	blood	**Ab34**	sputum	**Ab61**	Wound
**Ab8**	blood	**Ab35**	sputum	**Ab62**	Wound
**Ab9**	blood	**Ab36**	sputum	**Ab63**	Wound
**Ab10**	blood	**Ab37**	sputum	**Ab64**	Wound
**Ab11**	blood	**Ab38**	sputum	**Ab65**	Wound
**Ab12**	blood	**Ab39**	sputum	**Ab66**	Wound
**Ab13**	blood	**Ab40**	sputum	**Ab67**	Wound
**Ab14**	blood	**Ab41**	sputum	**Ab68**	Wound
**Ab15**	blood	**Ab42**	sputum	**Ab69**	Wound
**Ab16**	blood	**Ab43**	sputum	**Ab70**	Wound
**Ab17**	blood	**Ab44**	sputum	**Ab71**	Wound
**Ab18**	blood	**Ab45**	sputum	**Ab72**	Wound
**Ab19**	blood	**Ab46**	sputum	**Ab73**	Wound
**Ab20**	blood	**Ab47**	sputum	**Ab74**	Wound
**Ab21**	blood	**Ab48**	sputum	**Ab75**	Wound
**Ab22**	blood	**Ab49**	sputum	**Ab76**	Wound
**Ab23**	blood	**Ab50**	sputum	**Ab77**	Urine
**Ab24**	blood	**Ab51**	sputum	**Ab78**	Urine
**Ab25**	blood	**Ab52**	sputum	**Ab79**	Urine
**Ab26**	blood	**Ab53**	sputum	**Ab80**	Urine
**Ab27**	blood	**Ab54**	sputum		

Supplementary Table S2. Resistance pattern of *A. baumannii* clinical isolates and standard strains *A. baumannii* ATCC 19606 and 17978 to different antimicrobial agents.

Isolate code	Antimicrobial agents													Resistance pattern
	β-Lactams						Quinolones		Aminoglycosides		Sulfa drug	Tetracyclines		
	CAZ	FEP	CTX	TZP	SAM	IPM	CIP	LEV	AK	CN	SXT	DO	MIN	XDR isolate
Ab1	R	R	R	R	R	R	R	R	S	R	R	R	R	Yes
Ab2	R	R	R	R	R	R	R	R	R	R	R	S	R	Yes
Ab3	R	R	R	R	R	R	R	R	R	R	R	S	S	Yes
Ab4	R	R	R	R	R	R	R	R	R	R	R	S	S	Yes
Ab5	R	R	R	R	R	R	R	R	R	R	R	S	R	Yes
Ab6	R	R	R	R	R	R	R	R	R	R	R	R	R	Yes
Ab7	R	R	R	R	R	R	R	R	R	R	R	R	R	Yes
Ab8	R	R	R	R	R	R	R	R	R	R	R	R	I	Yes
Ab9	R	R	R	R	R	R	R	R	R	R	R	S	S	Yes
Ab10	R	R	R	R	R	R	R	R	R	R	R	S	S	Yes
Ab11	R	R	R	R	R	R	R	R	R	R	R	S	S	Yes
Ab12	R	R	R	R	R	R	R	R	R	R	R	S	S	Yes
Ab13	R	R	R	R	R	R	R	R	R	R	R	S	S	Yes
Ab14	R	R	R	R	R	R	R	R	R	R	R	S	S	Yes
Ab15	R	R	R	R	R	R	R	R	R	R	R	S	S	Yes
Ab16	R	R	R	R	R	R	R	R	R	R	R	R	R	Yes
Ab17	R	R	R	R	R	R	R	R	R	R	I	S	S	Yes
Ab18	R	R	R	R	R	R	R	R	R	R	R	R	R	Yes
Ab19	R	R	R	R	R	R	R	R	R	R	R	R	R	Yes
Ab20	R	R	R	R	R	R	R	R	R	S	R	S	S	Yes
Ab21	R	R	R	R	R	R	R	R	R	R	R	S	R	Yes
Ab22	R	R	R	R	R	R	R	R	R	R	R	R	I	Yes
Ab23	R	R	R	R	R	R	R	R	R	R	R	R	R	Yes
Ab24	R	R	R	R	R	R	R	R	R	S	R	S	S	Yes
Ab25	R	R	R	R	R	R	R	R	R	R	R	R	R	Yes
Ab26	R	R	R	R	R	R	R	R	R	R	R	S	S	Yes
Ab27	R	R	R	R	R	R	R	R	R	R	R	S	S	Yes
Ab28	R	R	R	R	R	R	R	R	R	R	R	S	R	Yes
Ab29	R	R	R	R	R	R	R	R	R	R	R	R	R	Yes
Ab30	R	R	R	R	R	R	R	R	R	R	R	S	R	Yes
Ab31	R	R	R	R	R	R	R	R	R	R	R	I	R	Yes
Ab32	R	R	R	R	R	R	R	R	R	R	R	S	I	Yes
Ab33	R	R	R	R	R	R	R	R	R	R	R	S	R	Yes
Ab34	R	R	R	R	R	R	R	R	R	R	R	I	R	Yes
Ab35	R	R	R	R	R	R	R	R	R	R	R	S	S	Yes
Ab36	R	R	R	R	R	R	R	R	R	R	R	S	I	Yes
Ab37	R	R	R	R	R	R	R	R	R	R	R	S	S	Yes
Ab38	R	R	R	R	R	R	R	R	R	R	R	S	R	Yes
Ab39	R	R	R	R	R	R	R	R	R	R	R	S	R	Yes
Ab40	R	R	R	R	R	R	R	R	R	R	R	I	R	Yes
Ab41	R	R	R	R	R	R	R	R	R	I	R	R	R	Yes
Ab42	R	R	R	R	R	R	R	R	R	I	R	R	R	Yes
Ab43	R	R	R	R	R	R	R	R	R	R	R	S	S	Yes
Ab44	R	R	R	R	R	R	R	R	R	R	R	R	R	Yes
Ab45	R	R	R	R	R	R	R	R	R	R	R	R	R	Yes
Ab46	R	R	R	R	S	S	S	S	S	S	R	S	S	No
Ab47	R	R	R	R	R	R	R	R	R	R	R	S	S	Yes
Ab48	R	R	R	R	R	R	R	R	R	R	R	R	S	Yes
Ab49	R	R	R	R	R	R	R	R	R	R	R	R	R	Yes
Ab50	R	R	R	R	R	R	R	R	R	R	R	R	R	Yes
Ab51	R	R	R	R	R	R	R	R	R	R	R	S	S	Yes
Ab52	R	R	R	R	R	R	R	R	R	R	R	R	R	Yes
Ab53	R	R	R	R	R	R	R	R	R	R	R	R	R	Yes
Ab54	R	R	R	R	R	R	R	R	R	R	R	R	R	Yes

**Ab55**	**R**	**R**	**R**	**R**	**R**	**R**	**R**	**R**	**R**	**R**	**R**	**S**	**S**	**Yes**
**Ab56**	**R**	**R**	**R**	**R**	**R**	**R**	**R**	**R**	**R**	**R**	**R**	**S**	**S**	**Yes**
**Ab57**	**R**	**R**	**R**	**R**	**R**	**R**	**R**	**R**	**R**	**R**	**R**	**R**	**R**	**Yes**
**Ab58**	**R**	**S**	**S**	**S**	**S**	**S**	**S**	**S**	**S**	**S**	**S**	**S**	**S**	**No**
**Ab59**	**R**	**R**	**R**	**R**	**R**	**R**	**R**	**R**	**R**	**R**	**R**	**R**	**S**	**Yes**
**Ab60**	**R**	**R**	**R**	**R**	**R**	**R**	**R**	**R**	**R**	**R**	**R**	**R**	**S**	**Yes**
**Ab61**	**R**	**R**	**R**	**R**	**R**	**R**	**R**	**R**	**R**	**R**	**S**	**R**	**S**	**Yes**
**Ab62**	**R**	**R**	**R**	**R**	**R**	**R**	**R**	**R**	**R**	**R**	**S**	**R**	**S**	**Yes**
**Ab63**	**R**	**R**	**R**	**R**	**R**	**R**	**R**	**R**	**R**	**R**	**S**	**R**	**S**	**Yes**
**Ab64**	**R**	**R**	**R**	**R**	**R**	**R**	**R**	**R**	**R**	**R**	**S**	**R**	**S**	**Yes**
**Ab65**	**R**	**R**	**R**	**R**	**R**	**R**	**R**	**R**	**R**	**R**	**S**	**R**	**S**	**Yes**
**Ab66**	**R**	**R**	**R**	**R**	**R**	**R**	**R**	**R**	**R**	**R**	**R**	**R**	**R**	**Yes**
**Ab67**	**R**	**R**	**R**	**R**	**R**	**R**	**R**	**R**	**R**	**R**	**R**	**R**	**R**	**Yes**
**Ab68**	**R**	**R**	**R**	**R**	**R**	**R**	**R**	**R**	**R**	**R**	**R**	**R**	**R**	**Yes**
**Ab69**	**R**	**R**	**R**	**R**	**R**	**R**	**R**	**R**	**R**	**R**	**S**	**R**	**S**	**Yes**
**Ab70**	**R**	**R**	**R**	**R**	**R**	**R**	**R**	**R**	**R**	**R**	**R**	**R**	**R**	**Yes**
**Ab71**	**R**	**R**	**R**	**R**	**R**	**R**	**R**	**R**	**R**	**R**	**R**	**R**	**R**	**Yes**
**Ab72**	**R**	**R**	**R**	**R**	**R**	**R**	**R**	**R**	**R**	**R**	**S**	**R**	**S**	**Yes**
**Ab73**	**R**	**R**	**R**	**R**	**R**	**R**	**R**	**R**	**R**	**R**	**R**	**R**	**R**	**Yes**
**Ab74**	**R**	**R**	**R**	**R**	**R**	**R**	**R**	**R**	**R**	**R**	**R**	**R**	**R**	**Yes**
**Ab75**	**R**	**R**	**R**	**R**	**R**	**R**	**R**	**R**	**R**	**R**	**R**	**R**	**R**	**Yes**
**Ab76**	**R**	**R**	**R**	**R**	**R**	**R**	**R**	**R**	**R**	**R**	**S**	**R**	**S**	**Yes**
**Ab77**	**R**	**R**	**R**	**R**	**R**	**R**	**R**	**R**	**R**	**R**	**S**	**R**	**S**	**Yes**
**Ab78**	**R**	**R**	**R**	**R**	**R**	**R**	**R**	**R**	**R**	**R**	**R**	**R**	**R**	**Yes**
**Ab79**	**R**	**R**	**R**	**R**	**R**	**R**	**R**	**R**	**R**	**R**	**S**	**R**	**S**	**Yes**
**Ab80**	**R**	**R**	**R**	**R**	**R**	**R**	**R**	**R**	**S**	**R**	**S**	**R**	**S**	**Yes**
**ATCC 19606**	**R**	**R**	**R**	**R**	**R**	**S**	**S**	**S**	**S**	**S**	**R**	**S**	**S**	**No**
**ATCC 17987**	**S**	**S**	**S**	**S**	**S**	**S**	**S**	**S**	**S**	**S**	**R**	**S**	**S**	**No**
**Total number of resistant isolates (%)**	**80 (100)**	**79 (99)**	**79 (99)**	**79 (99)**	**78 (97.5)**	**78 (97.5)**	**78 (97.5)**	**78 (97.5)**	**76 (95)**	**74 (92.5)**	**67 (83.8)**	**44 (55)**	**39 (48.8)**	**78 (97.5)**

CAZ: ceftazidime, FEP: cefepime, CTX: cefotaxime, TZP: tazobactam/piperacillin, SAM: sulbactam /ampicillin, IPM: imipenem, CIP: ciprofloxacin, LEV: levofloxacin, AK: amikacin, CN: gentamicin, SXT: sulfamethoxazole/trimethoprim, DO: doxycycline, MIN: minocycline, XDR: extensively drug-resistant, **R**: resistant, **I**: intermediate, and **S**: sensitive.

Supplementary Table S3. Molecular characterization of resistance mechanisms of *A. baumannii* isolates and standard strains *A. baumannii* ATCC 19606 and 17978 to fluoroquinolones.

**Isolate code**	**Fluoroquinolone-resistance determining regions**				**Plasmid-mediated quinolone resistance genes (*qnrA, qnrB, qnrC, qnrD, qepA,* and *oqxAB* genes were absent in all isolates)**		
	***gyrA* gene**		***parC* gene**				
	**Detection**	**S83L Mutation**	**Detection**	**S80L Mutation**	***aac(6')-Ib* gene**	***qnrS* gene**	***qnrS+ aac(6')-Ib* genes**
**Ab1**	+	+	+	+	+	-	-
**Ab2**	+	+	+	+	+	-	-
**Ab3**	+	+	+	+	+	-	-
**Ab4**	+	+	+	+	+	-	-
**Ab5**	+	+	-	-	+	-	-
**Ab6**	+	+	+	+	-	-	-
**Ab7**	+	+	+	+	+	-	-
**Ab8**	-	-	+	+	+	-	-
**Ab9**	+	+	+	+	+	-	-
**Ab10**	+	+	+	+	-	-	-
**Ab11**	-	-	+	+	+	-	-
**Ab12**	+	+	+	+	+	-	-
**Ab13**	+	+	+	+	+	-	-
**Ab14**	+	+	+	+	+	-	-
**Ab15**	+	+	+	+	+	-	-
**Ab16**	+	+	+	+	-	-	-
**Ab17**	+	+	+	+	-	-	-
**Ab18**	+	+	+	+	+	-	-
**Ab19**	+	+	+	+	+	-	-
**Ab20**	+	+	+	+	+	-	-
**Ab21**	-	-	+	+	-	-	-
**Ab22**	+	+	+	+	+	-	-
**Ab23**	+	+	+	+	+	-	-
**Ab24**	+	+	+	+	-	-	-
**Ab25**	+	+	+	+	+	-	-
**Ab26**	+	+	+	+	+	-	-
**Ab27**	+	+	+	+	+	-	-
**Ab28**	+	+	+	+	+	-	-
**Ab29**	+	+	+	+	+	-	-
**Ab30**	+	+	+	+	+	-	-
**Ab31**	+	+	+	+	+	-	-
**Ab32**	+	+	+	+	+	-	-
**Ab33**	+	+	+	+	+	-	-
**Ab34**	+	+	-	-	+	-	-
**Ab35**	+	+	-	-	+	-	-
**Ab36**	+	+	+	+	-	-	-
**Ab37**	-	-	+	+	-	-	-
**Ab38**	-	-	+	+	+	-	-
**Ab39**	+	+	+	+	+	-	-
**Ab40**	+	+	+	+	+	-	-
**Ab41**	+	+	+	+	+	-	-
**Ab42**	+	+	+	+	+	-	-
**Ab43**	+	+	+	+	+	-	-
**Ab44**	+	+	+	+	+	-	-
**Ab45**	+	+	+	+	+	-	-
**Ab46**	+	+	+	+	+	-	-
**Ab47**	+	+	+	+	+	-	-

**Ab48**	+	+	+	+	+	-	-
**Ab49**	+	+	+	+	+	-	-
**Ab50**	+	+	+	+	+	-	-
**Ab51**	+	+	+	+	+	-	-
**Ab52**	+	+	+	+	+	-	-
**Ab53**	+	+	+	+	+	-	-
**Ab54**	+	+	+	+	+	-	-
**Ab55**	+	+	+	+	+	-	-
**Ab56**	+	+	+	+	-	-	-
**Ab57**	+	+	-	-	+	-	-
**Ab58**	-	-	+	+	+	-	-
**Ab59**	-	-	-	-	-	-	-
**Ab60**	+	+	+	+	+	+	+
**Ab61**	+	+	+	+	+	-	-
**Ab62**	+	+	+	+	+	-	-
**Ab63**	+	+	+	+	+	-	-
**Ab64**	-	-	+	+	+	-	-
**Ab65**	+	+	+	+	+	+	+
**Ab66**	+	+	+	+	+	-	-
**Ab67**	-	-	+	+	-	-	-
**Ab68**	+	+	+	+	+	-	-
**Ab69**	+	+	+	+	+	-	-
**Ab70**	+	+	+	+	+	-	-
**Ab71**	+	+	+	+	-	-	-
**Ab72**	+	+	+	+	-	-	-
**Ab73**	+	+	+	+	+	-	-
**Ab74**	+	+	+	+	+	-	-
**Ab75**	-	-	+	+	-	-	-
**Ab76**	-	-	+	+	-	-	-
**Ab77**	+	+	+	+	-	-	-
**Ab78**	+	+	+	+	-	-	-
**Ab79**	+	+	+	+	-	-	-
**Ab80**	+	+	+	+	-	-	-
**ATCC 19606**	+	-	+	-	-	-	-
**ATCC 17987**	+	-	+	-	-	-	-
**Total number of positive isolates (%)**	69 (86.3)	69 (86.3)	75 (93.8)	75 (93.8)	61 (76.3)	2 (2.5)	2 (2.5)
(+): Detected or present, (-): Not detected or absent, S80L: Ser80 to Leu and S83L: Ser83 to Leu.							

Supplementary Table S4. Primers used in this study.

Gene or DNA region		Primer	Nucleotide Sequence (5' - 3')	Annealing temp (°C)	Amplicon size (bp)	Reference
recA gene of *Acinetobacter* genus		*p-rA1*	CCTGAATCTTCTGGTAAAAC	50	425	**1**
		p-rA2	GTTTCTGGGCTGCCAAACATTAC			
The intergenic spacer region region of *A. baumannii* (*16S-23SrRNA ITS*)		*p-Ab-ITSF*	CATTATCACGGTAATTAGTG	50	208	**2**
		p-Ab-ITSB	AGAGCACTGTGCACTTAAG			
FQRDRs	*gyrA*	*gyrA-F*	AAATCTGCCCGTGTCGTTGGT	56	343	**3**
		gyrA-R	GCCATACCTACGGCGATACC			
	parC	*parC-F*	AAACCTGTTCAGCGCCGCATT	54	327	**4**
		parC-R	AAAGTTGTCTTGCCATTCACT			
PMQR genes	*qnrA*	*qnrA-F*	AGAGGATTTCTCACGCCAGG	57	579	**5**
		qnrA-R	TGCCAGGCACAGATCTTGAC			
	qnrB	*qnrB-F*	GGMATHGAAATTCGCCACTG	56	263	
		qnrB-R	TTYGCBGYYCGCCAGTCGAA			
	qnrS	*qnrS-F*	GCAAGTTCATTGAACAGGGT	53	427	
		qnrS-R	TCTAAACCGTCGAGTTCGGCG			
	qnrC	*qnrC-F*	GGGTTGTACATTTATTGAATCG	51	308	**6**
		qnrC-R	CACCTACCCATTTATTTTCA			
	qnrD	*qnrD-F*	ATGGAAAAGCACTTTATCAA	50	645	**7**
		qnrD-R	TTATCGGTGAACAATAACAC			
	aac(6')-Ib	*aac(6')-Ib-F*	CTTGCGATGCTCTATGAGTGG	57	480	**8**
		aac(6')-Ib-R	GAATGCCTGGCGTGTTTGAA			
Efflux pump genes	*oqxAB*	*oqxAB-F*	GACGACAAAGCGGTACTGAC	56	448	**7**
		oqxAB-R	GTCTCGGCAATCACTTTCGG			
	qepA	*qepA-F*	TCTACGGGCTCAAGCAGTTG	57	380	**8**
		qepA-R	ACAGCGAACCGATGACGAAG			

Temp: temperature, F: forward, R: reverse, bp: base pair, FQRDRs: fluoroquinolone resistance-determining regions, and PMQR: plasmid-mediated quinolone resistance.

Supplementary Table S5. Effect of CCCP on the MICs of plain PCL nanoparticles, CIP, and LEV against representative fluoroquinolone (FQ)-resistant and extensively drug-resistant (XDR) *A. baumannii* isolates and standard strains *A. baumannii* ATCC 19606 and 17978.

Isolate code	MIC* (µg/ml)				MDF
	Plain	CIP	CIP + CCCP	Plain + CIP + CCCP	
**Ab29**	-	32	32	32	1
**Ab30**	-	32	8	8	4
**Ab36**	-	32	32	32	1
**Ab60**	-	128	16	16	8
**Ab65**	-	64	64	64	1
**Ab71**	-	64	64	64	1
**Ab72**	-	64	16	16	4
**Ab77**	-	64	64	64	1
**ATCC 19606**	-	0.5	0.5	0.5	1
**ATCC 17987**	-	0.25	0.25	0.25	1
**Isolate code**	**MIC* (µg/ml)**				**MDF**
	**Plain**	**LEV**	**LEV + CCCP**	**Plain + LEV + CCCP**	
**Ab29**	-	8	8	8	1
**Ab30**	-	8	2	2	4
**Ab36**	-	8	8	8	1
**Ab60**	-	16	4	4	4
**Ab65**	-	16	16	16	1
**Ab71**	-	8	8	8	1
**Ab72**	-	8	2	2	4
**Ab77**	-	8	8	8	1
**ATCC 19606**	-	0.5	0.5	0.5	1
**ATCC 17987**	-	0.25	0.25	0.25	1

MIC: minimum inhibitory concentration, CIP: ciprofloxacin, LEV: levofloxacin, PCL: poly Ɛ-caprolactone, Plain: plain PCL nanoparticles, MDF: MIC decrease factor, and CCCP: carbonyl-cyanide-m-chlorophenylhydrazone.
***:** The mean MIC value was reported after experiments were carried out in duplicate.

Supplementary Table S6. Distribution of *A. baumannii* isolates and standard strains *A. baumannii* ATCC 19606 and 17978 according to biofilm formation.

Isolate code	Biofilm formation	OD*	Isolate code	Biofilm formation	OD*
Ab1	N	0.022	Ab41	S	0.402
Ab2	W	0.095	Ab42	S	0.322
Ab3	S	0.522	Ab43	W	0.128
Ab4	S	0.489	Ab44	M	0.27
Ab5	S	0.451	Ab45	S	0.341
Ab6	S	0.422	Ab46	W	0.121
Ab7	W	0.115	Ab47	S	0.364
Ab8	S	0.391	Ab48	S	0.373
Ab9	N	0.028	Ab49	S	0.391
Ab10	S	0.371	Ab50	S	0.322
Ab11	W	0.132	Ab51	W	0.123
Ab12	S	0.42	Ab52	S	0.331
Ab13	S	0.373	Ab53	S	0.322
Ab14	W	0.123	Ab54	S	0.385
Ab15	S	0.521	Ab55	S	0.391
Ab16	S	0.432	Ab56	S	0.372
Ab17	S	0.367	Ab57	S	0.356
Ab18	S	0.395	Ab58	M	0.19
Ab19	S	0.421	Ab59	M	0.272
Ab20	S	0.478	Ab60	S	0.45
Ab21	S	0.341	Ab61	M	0.202
Ab22	S	0.357	Ab62	M	0.192
Ab23	S	0.364	Ab63	M	0.183
Ab24	W	0.131	Ab64	M	0.173
Ab25	S	0.398	Ab65	S	0.319
Ab26	S	0.38	Ab66	M	0.265
Ab27	S	0.351	Ab67	M	0.269
Ab28	S	0.284	Ab68	W	0.082
Ab29	S	0.371	Ab69	S	0.402
Ab30	S	0.541	Ab70	M	0.27
Ab31	S	0.356	Ab71	S	0.396
Ab32	S	0.381	Ab72	S	0.47
Ab33	S	0.394	Ab73	M	0.269
Ab34	S	0.322	Ab74	M	0.232
Ab35	S	0.341	Ab75	M	0.217
Ab36	S	0.542	Ab76	M	0.267
Ab37	M	0.25	Ab77	S	0.328
Ab38	S	0.361	Ab78	M	0.247
Ab39	W	0.125	Ab79	W	0.123
Ab40	S	0.375	Ab80	M	0.212
ATCC 19606	S	0.301	ATCC 17987	S	0.335
Total Number (%) of strong (S), moderate (M), week (W), and non (N)- biofilm forming isolates are 50 (62.5%), 17 (21.3%), 11 (13.8%) and 2 (2.5%) isolates, respectively.					

*: The values represent the mean ± SD of each isolate's optical density (OD) of four wells.

Supplementary Table S7. Ingredients of the prepared nanoparticles.

Parameter \ Formula			F_{CIP}	F_{LEV}	Plain NPs
Ingredients % (w/v)	O	PCL	1.5	1.5	1.5
	W_1	CIP	2	---	---
		LEV	---	2	---
	W_2	PVA	0.5	0.5	0.5

CIP: ciprofloxacin, LEV: levofloxacin, F_{CIP}: CIP-loaded nanoparticles, F_{LEV}: LEV-loaded nanoparticles, Plain NPs: plain PCL nanoparticles, O: organic phase, W_1: internal aqueous phase, W_2: external aqueous phase, PCL: poly Ɛ-caprolactone, and PVA: polyvinyl alcohol.

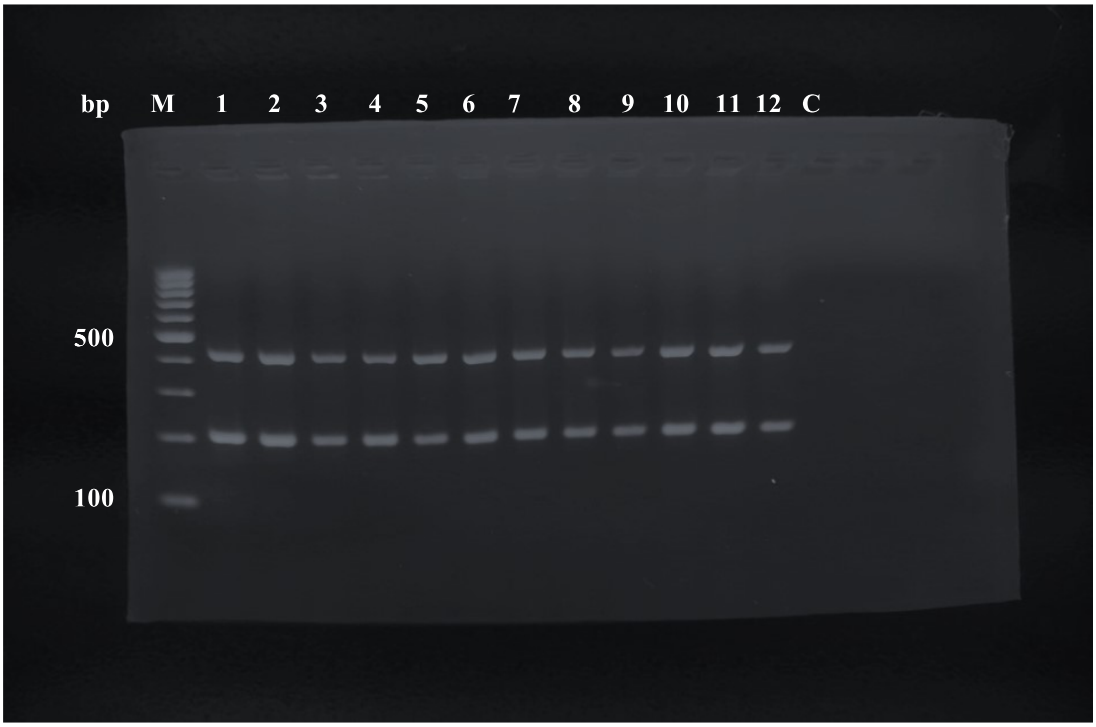

Supplementary Fig. S1. Multiplex PCR for detection of *recA* gene (characteristic of the *Acinetobacter* genus) (425 bp) and *16S-23S rRNA* gene intergenic spacer (*ITS)* region (specific for *A. baumannii* spp.) (208 bp) in representative isolates of *A. baumannii*. Lane M: 100 bp DNA ladder, Lanes 1-10: positive isolates of *A. baumannii* (Ab1-10), Lanes 11 and 12 represent *A. baumannii* ATCC 19606 and 17978, respectively, Lane C: negative control (PCR reaction without DNA template), and bp: base pairs.

a

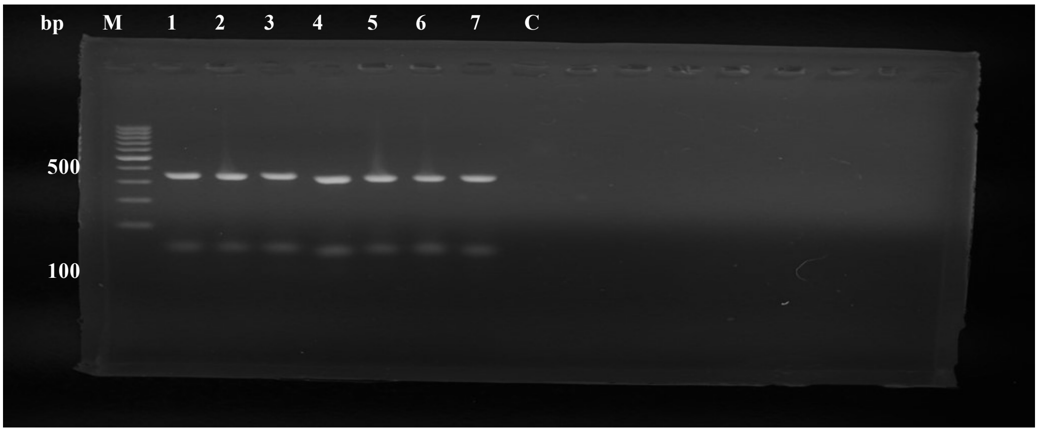

343 bp

b

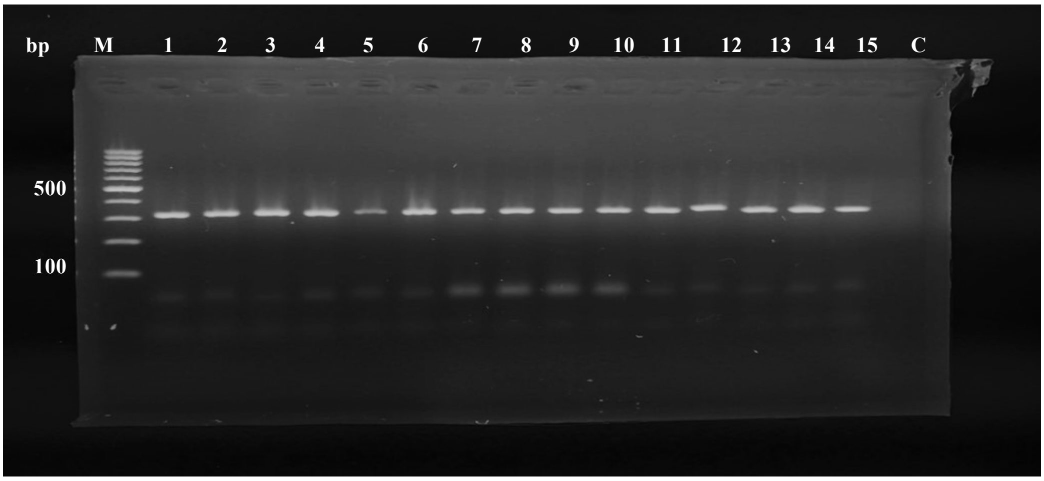

327 bp

Supplementary Fig. S2. Detection of mutation in the fluoroquinolone resistance-determining regions (FQRDRs) after *HinfI* digestion of the PCR products of *gyrA* and *parC* genes. **a)** Mutation in the FQRDRs of *gyrA* is indicated by undigested fragment (343 bp) and Lanes 1-15 represent *A. baumannii* (Ab20-34) and Lanes 1-7 represent undigested fragments of *A. baumannii* (Ab1-7). **b)** Mutation in the FQRDRs of *parC* is indicated by undigested fragment (327 bp) and Lanes 1-15 represent undigested fragments of *A. baumannii* (Ab1-15). Lane M: 100 bp DNA ladder, Lane C: negative control (PCR reaction without DNA template) and bp: base pairs.

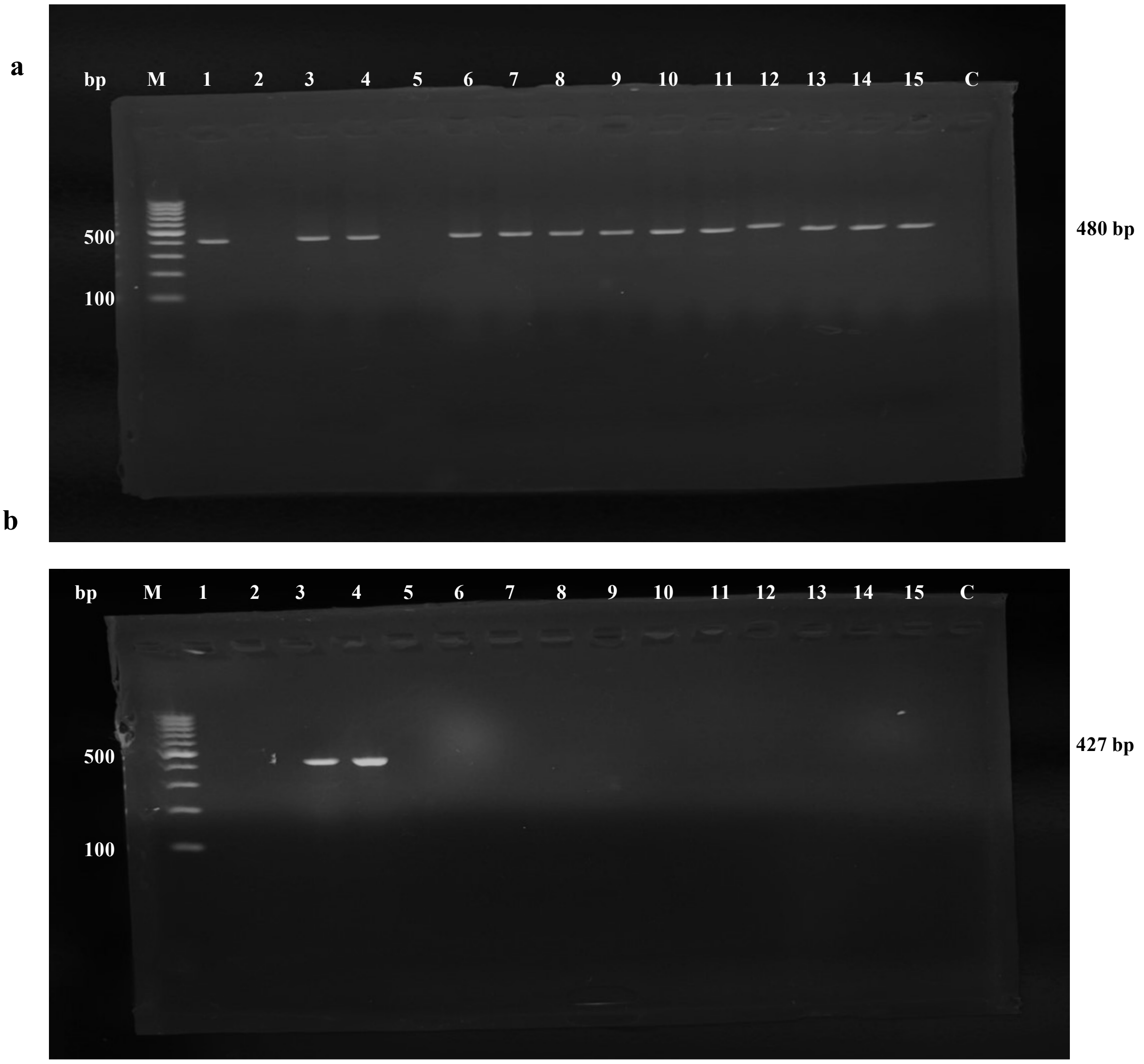

Supplementary Fig. S3. Detection of the plasmid-mediated quinolone resistance (PMQR) genes of PCR products of *aac(6')-Ib* and *qnrS* genes. **a)** *aac(6')-Ib* gene (480 bp) and Lanes 1-15 represent *A. baumannii* (Ab20-34), **b)** *qnrS* gene (427 bp) and Lanes 1-15 represent *A. baumannii* (Ab58-60 and Ab65-76). Lane M: 100 bp DNA ladder, Lane C: negative control (PCR reaction without DNA template) and bp: base pairs.

References:

1 Krawczyk, B., Lewandowski, K. & Kur, J. Comparative studies of the *Acinetobacter* genus and the species identification method based on the *recA* sequences. *Mol Cell Probes* **16**, 1-11, doi:10.1006/mcpr.2001.0388 (2002).

2 Chen, T. L. *et al.* Comparison of one-tube multiplex PCR, automated ribotyping and intergenic spacer (ITS) sequencing for rapid identification of *Acinetobacter baumannii. Clin Microbiol Infect* **13**, 801-806, doi:10.1111/j.1469-0691.2007.01744.x (2007).

3 Vila, J., Ruiz, J., Goñi, P., Marcos, A. & Jimenez de Anta, T. Mutation in the *gyrA* gene of quinolone-resistant clinical isolates of *Acinetobacter baumannii*. *Antimicrob Agents Chemother* **39**, 1201-1203, doi:10.1128/aac.39.5.1201 (1995).

4 Vila, J., Ruiz, J., Goñi, P. & Jimenez de Anta, T. Quinolone-resistance mutations in the topoisomerase IV *parC* gene of *Acinetobacter baumannii*. *J Antimicrob Chemother* **39**, 757-762, doi:10.1093/jac/39.6.757 (1997).

5 Cattoir, V., Poirel, L., Rotimi, V., Soussy, C. J. & Nordmann, P. Multiplex PCR for detection of plasmid-mediated quinolone resistance *qnr* genes in ESBL-producing enterobacterial isolates. *J Antimicrob Chemother* **60**, 394-397, doi:10.1093/jac/dkm204 (2007).

6 Kim, H. B. *et al.* Prevalence of Plasmid-Mediated Quinolone Resistance Determinants over a 9-Year Period. *Antimicrobial Agents and Chemotherapy* **53**, 639-645, doi:doi:10.1128/AAC.01051-08 (2009).

7 Rodríguez-Martínez, J. M. *et al.* Plasmid-mediated quinolone resistance: Two decades on. *Drug Resistance Updates* **29**, 13-29, doi:https://doi.org/10.1016/j.drup.2016.09.001 (2016).

8 Hamed, S. M. *et al.* Plasmid-Mediated Quinolone Resistance in Gram-Negative Pathogens Isolated from Cancer Patients in Egypt. *Microb Drug Resist* **24**, 1316-1325, doi:10.1089/mdr.2017.0354 (2018).
